# Arabidopsis Mitochondrial Transcription Termination Factor mTERF2 Promotes Splicing of Group IIB Introns

**DOI:** 10.3390/cells10020315

**Published:** 2021-02-03

**Authors:** Kwanuk Lee, Dario Leister, Tatjana Kleine

**Affiliations:** Plant Molecular Biology (Botany), Department Biology I, Ludwig-Maximilians-Universität München, 82152 Planegg-Martinsried, Germany; kwanuk.lee@biologie.uni-muenchen.de (K.L.); leister@lmu.de (D.L.)

**Keywords:** Arabidopsis, chloroplast, development, mTERF, plastid gene expression, splicing

## Abstract

Plastid gene expression (PGE) is essential for chloroplast biogenesis and function and, hence, for plant development. However, many aspects of PGE remain obscure due to the complexity of the process. A hallmark of nuclear-organellar coordination of gene expression is the emergence of nucleus-encoded protein families, including nucleic-acid binding proteins, during the evolution of the green plant lineage. One of these is the mitochondrial transcription termination factor (mTERF) family, the members of which regulate various steps in gene expression in chloroplasts and/or mitochondria. Here, we describe the molecular function of the chloroplast-localized mTERF2 in *Arabidopsis thaliana*. The complete loss of mTERF2 function results in embryo lethality, whereas directed, microRNA (*amiR*)-mediated knockdown of *MTERF2* is associated with perturbed plant development and reduced chlorophyll content. Moreover, photosynthesis is impaired in *amiR-mterf2* plants, as indicated by reduced levels of photosystem subunits, although the levels of the corresponding messenger RNAs are not affected. RNA immunoprecipitation followed by RNA sequencing (RIP-Seq) experiments, combined with whole-genome RNA-Seq, RNA gel-blot, and quantitative RT-PCR analyses, revealed that mTERF2 is required for the splicing of the group IIB introns of *ycf3* (intron 1) and *rps12*.

## 1. Introduction

Plastids of the group Plantae arose more than one-billion years ago from a single endosymbiosis event, in which a heterotrophic protist was engulfed and retained a cyanobacterium in its cytoplasm [1]. During evolution, plastids lost most of their genes to the nucleus [2] and they have only retained a reduced genome of approximately 120 loci. Consequently, both nucleus- and plastid-encoded components are required for processes that take place in chloroplasts, including the formation of the photosystems, the Rubisco complex, and plastid ribosomes. In consequence, the lack of nucleus-encoded proteins necessary for plastid gene expression (PGE) often results in pale green plants and even seedling- or embryo-lethality [3], which underlines the importance of PGE for plant development and photosynthesis. However, the regulation of PGE is only partially understood, because the PGE system in plants is far more complex than that of its cyanobacterial progenitor [4]. Once transcribed, organellar RNAs can undergo various post-transcriptional modification events, such as splicing, trimming, and editing [5,6,7]. With one exception (the plastid-encoded maturase MatK), all of the proteins that are needed for organellar RNA maturation in land plants are encoded in the nucleus [6]. These proteins encompass members of the pentatricopeptide repeat (PPR), half-a-tetratricopeptide (HAT), and octotricopeptide repeat (OPR) families, as well as ‘mitochondrial transcription termination factor’ (mTERF) proteins [5]. mTERFs have been identified in both metazoans and plants, and their modular architecture is characterized by repeats of a 30-amino-acid motif, the so-called MTERF motif [8]. The number and composition of these motifs, as well as the remaining sequences, vary widely within the family. Although the family received its name from its founding member—human mTERF1, which was proposed to act as transcription termination factor in mitochondrial extracts [9]—this function is now in dispute [10]. Thus, three of the four mammalian mTERFs do not actually terminate transcription, but they appear to participate in polar replication fork pausing [11], antisense transcription termination, and ribosome biogenesis [10], while the role of mTERF2 remains to be clarified. 

The mTERF family has expanded to approximately 30 members during the evolution of land plants [12], and most of the 35 *Arabidopsis thaliana* (*A. thaliana*) mTERF proteins are targeted to mitochondria and/or chloroplasts [13]. The molecular functions of five of them (mTERF4, -5, -6, -8, and -15) have been investigated in more detail [10,14,15,16]. *A. thaliana* mTERF5 [17,18] and mTERF8 [19] bind to different dsDNA sequences that are associated with the *psbEFLJ* polycistron, which encodes subunits of photosystem II (PSII). It was suggested that mTERF8 terminates transcription [19] and, according to Ding et al. [17], mTERF5 acts as a transcriptional pausing factor to induce pausing of the plastid-encoded RNA polymerase (PEP) complex. Métegnier et al. [18] identified the 5′ region of the *ndhA* gene as a second binding site for mTERF5, and proposed that mTERF5 stimulates both the transcription and stabilization of the 5′-ends of processed *psbE* and *ndhA* transcripts. The mTERF6 protein is required for the maturation of a chloroplast isoleucine transfer RNA (*trnI.2*) [20] and, more recently, a function in transcription termination downstream of the plastid *rpoA* polycistron was reported for this mTERF member [21].

The results that are summarized above already indicate that plant mTERFs have evolved to bind DNA as well as RNA, and perform diverse functions, including intron splicing [13,22,23]. Based on their structures, splicing, and mobilization mechanisms, introns are classified into groups I, II, and III [24,25]. Group II introns, in turn, were initially arranged into the three groups IIA, IIB, and IIC, based on RNA sequence and secondary structure [26,27]. The *A. thaliana* plastid genome harbors one group I intron, eight group IIA, and 12 group IIB introns. In fact, maize mTERF4, an ortholog of the *A. thaliana* mTERF protein variously named BELAYA SMERT (BSM, [13]), RUGOSA2 (RUG2, [28]), or mTERF4 [12], primarily promotes the splicing of the group IIA introns of *trnI.2*, *trnA*, *rpl2,* and *atpF*, together with the second intron of *ycf3*, a group IIB intron. Its intron splicing function might be conserved in *A. thaliana* mTERF4, because there is strong evidence that this protein is required for the removal of at least the second intron of *clpP* [13], a group IIA intron that is not found in maize. In light of the role of mTERF4 in chloroplast intron splicing [13,22], the mitochondrial splicing events in the *mterf15* mutant and complemented plants have been investigated [23]. In *mterf15*, the splicing of *nad2* intron 3 is significantly inhibited, a defect that is fully rescued in the complemented plants, which suggests that mTERF15 is involved in the splicing of the third intron in the mitochondrial *nad2* transcript, but, here again, binding to the target remains to be demonstrated.

In this work, we shed light on the molecular function of chloroplast-localized mTERF2. Because knock-out *mterf2* mutant plants are embryo lethal, artificial microRNA lines were generated in order to study the biological roles(s) of mTERF2 in seedlings and adult plants. In *mterf2* knock-down mutant plants, photosynthesis and growth are impaired, and flowering is delayed. We identified RNA targets of mTERF2 and showed that the protein is required for the splicing of the group IIB introns of *ycf3* (intron 1) and *rps12*.

## 2. Materials and Methods

### 2.1. Plant Material and Growth Conditions 

The mutants *mterf2-1* (SALK_141541), *mterf2-2* (SALK_017427), and *mterf2-3* (SALK_100040) were identified in the SIGnAL database (Alonso et al., 2003). Insertions were confirmed with the primers that are listed in Supplementary Table S1 and all of the mutants are in the Col-0 background. *A. thaliana* plants were grown on potting soil (Stender) under controlled greenhouse conditions on an 16/8-h light/dark cycle; daylight was supplemented with illumination from HQI Powerstar 400W/D (Osram, Munich, Germany), providing a total fluence of approximately 120 μmol photons m^−2^ s^−1^ on leaf surfaces. Wild-type (Col-0) and mutant seeds were sterilized with 70% [*v*/*v*] ethanol and the surface-sterilized seeds were stratified for at least two days at 4 °C. Where indicated, the seedlings were grown on autoclaved half-strength MS medium containing 1.0% (*w*/*v*) sucrose, 0.05% MES (pH 5.8), and 0.8 % plant agar (Duchefa, Haarlem, The Netherlands) at 22 °C under illumination that was provided by white fluorescent lamps (100 μmol photons m^−2^ s^−1^).

### 2.2. Nucleic Acid Extraction

For DNA isolation, the leaf tissue was homogenized in extraction buffer containing 200 mM Tris-HCl (pH 7.5), 25 mM NaCl, 25 mM EDTA, and 0.5% (*w*/*v*) sodium dodecyl sulfate (SDS). After centrifugation, DNA was precipitated from the supernatant by adding isopropyl alcohol. After washing with 70% (*v*/*v*) ethanol, the DNA was dissolved in distilled water. For RNA isolation, frozen tissue was ground in liquid nitrogen. The total RNA was extracted with the Direct-zol™ RNA Miniprep Plus Kit (Zymo Research, Irvine, CA, USA), according to the manufacturer’s instructions. The RNA quality (A260/A280 ratio > 1.8, A260/230 ratio > 1.8) and concentration were assessed by agarose gel electrophoresis and spectrophotometry, respectively. The isolated RNA was kept at -80 °C prior to use.

### 2.3. cDNA Synthesis and Real-Time PCR Analysis

For complementary DNA (cDNA) synthesis, 1-μg aliquots of total RNA were employed in order to synthesize cDNA while using the iScript cDNA Synthesis Kit (Bio-Rad, Hercules, CA, USA). Real-time quantitative PCR experiments were performed on a Bio-Rad iQ5 real-time instrument with the iQ SYBR Green Supermix (Bio-Rad, Hercules, CA, USA) with a standard thermal profile (95 °C for 5 min., 40 cycles of 95 °C for 10 s, 55 °C for 30 s, and 72 °C for 20 s). Each sample was quantified in triplicate and, as an internal control, normalized to the values of *AT4G36800*, which codes for RUB1-conjugating enzyme (RCE1).

### 2.4. Generation of amiRNA-Mediated Knockdown Lines, GUS and c-myc Fusion Constructs

Knockdown *mterf2–amiR1* and *amiR2* mutant plants were generated while using an artificial microRNA (amiRNA)-mediated knockdown technique [29]. The Web MicroRNA Designer program (http://wmd3.weigelworld.org/) was applied in order to generate two amiRNA constructs (amiR1 and amiR2) targeting different regions of the *MTERF2* (*AT2G21710*) gene. The amiR constructs were generated, as described [30], while using the primers provided in Supplementary Table S1. The individual T1 seeds were selected by GFP fluorescence, and independent T3 and T4 transgenic lines were used for phenotypic analysis. The knockdown levels of T3 *mterf2–amiR* lines were confirmed by reverse transcription (RT)-PCR and real-time PCR with the gene-specific primers that are listed in Supplementary Table S1. In order to investigate tissue-specific expression patterns of *MTERF2* with the GUS reporter and produce a c-myc-tagged mTERF2 protein, the genomic DNA fragment encompassing the putative promoter and coding region of *MTERF2* were separately amplified while using the gene-specific primers that are listed in Supplementary Table S1. Each PCR product was then cloned into the pDONR207 vector via the BP reaction (Thermo Fisher Scientific, Waltham, MA, USA). Subsequent subcloning steps, into the pGWB533 vector for fusion with the GUS reporter gene and the pGWB517 vector for C-terminal fusion with the 4x c-myc tag, were carried out via the LR reaction (Thermo Fisher Scientific, Waltham, MA, USA). The resulting constructs were transformed into the *Agrobacterium tumefaciens* GV3101 strain, and then introduced into Arabidopsis Col-0 via floral dip [31].

### 2.5. Expression and Intracellular Localization of Fluorescence Fusions

The coding region of *MTERF2* was amplified from cDNA by PCR with the gene-specific primers that are listed in Supplementary Table S1. The PCR product was cloned with GATEWAY technology into the pB7FWG2 vector in order to generate a fusion with eGFP under the regulation of the Cauliflower mosaic virus 35S promoter. Subcellular localization analysis of fused proteins was conducted in protoplasts that were isolated from wild-type (Col-0). The protoplasts were imaged with a Fluorescence AxioImager microscope (Zeiss, Oberkochen, Germany) after transformation, as described in [32]. Fluorescence was excited with the X-Cite Series 120 fluorescence lamp (EXFO, Quebec, QC, Canada) and the images were collected at 500–550 nm (eGFP fluorescence) and 670–750 nm (Chl autofluorescence). For co-transformation experiments, RAP-RFP that was generated in [33] was used as a marker for chloroplast nucleoids.

### 2.6. Leaf Pigment Analyses

For chlorophyll extraction, approximately 10–30 mg of leaf tissue from six-day-old and four-week-old plants, respectively, was ground in liquid nitrogen and then incubated in the presence of 80% (*v*/*v*) acetone. After the removal of cell debris by centrifugation, absorption was measured with an Ultrospec 3100 pro spectrophotometer (Amersham Biosciences, Freiburg, Germany). The pigment concentrations were calculated following [34]. 

### 2.7. Chlorophyll Fluorescence Analysis

In vivo Chl *a* fluorescence of whole plants was recorded using an ImagingPAM Chl fluorometer (Heinz Walz GmbH, Effeltrich, Germany). Dark-adapted (20–30 min.) plants were exposed to a pulsed, blue measuring beam (1 Hz, intensity 4; *F_0_*) and a saturating light flash (intensity 4) to obtain *Fv/Fm* = *(Fm - F0)/Fm* (maximum quantum yield of PSII).

### 2.8. Protein Isolation and Immunoblot Analyses 

Samples (approximately 100 mg) of six-day-old seedlings were ground in liquid nitrogen and the total proteins were solubilized in protein extraction buffer (125 mM Tris, 1% SDS, 10% [*v*/*v*] glycerol, 0.05 M sodium metabisulfite) containing 1 mM phenylmethylsulfonyl fluoride (PMSF) and protease inhibitor cocktail (Roche, Mannheim, Germany), as described in [35]. Cell debris was eliminated by centrifugation at 16,000x g for 20–30 min., and the supernatant was transferred to a new tube; 5x SDS loading buffer (60 mM Tris-HCl, pH 6.8, 25 % (*v*/*v*) glycerol, 2% (*w*/*v*) SDS, 14.4 mM 2-mercapto ethanol, and 0.1% (*w*/*v*) bromophenol blue) was then added and the solution was heated at 95 °C for 10 min. Equal amounts of total proteins were fractionated on 10–12% SDS-PA gels and then transferred to polyvinylidene fluoride membranes (PVDF) (Millipore, Darmstadt, Germany) while using the semidry method (Turbo transfer system (Bio-Rad). The primary antibodies that were directed against PsbD (1:5,000), PsbO (1:5,000), PsaA (1:1,000), PsaD (1:1,000), cyt*b_6_* (1:10,000), Atpβ (1:5,000), RbcL (1:10,000), and Actin (1:1,000) used in this study were purchased from Agrisera. The signals were detected with the PierceECL Western Blotting Kit (Thermo Fisher Scientific, Waltham, MA, USA), recorded using an ECL reader system (Fusion FX7; PeqLab, Life Science, VWR, Ismaning, Germany), and quantified using ImageJ [36].

### 2.9. Chloroplast Fractionation

Intact chloroplasts from three-week-old Arabidopsis plants were prepared, as described in [37] with the following minor modifications of centrifuge speeds and times. The chloroplasts were pelleted at 800 g for 8 min. (brake off, 4 °C), resuspended in HS buffer, loaded onto a 40%/85% Percoll gradient, and then centrifuged at 600 g for 20 min. (brake off, 4 °C). The intact chloroplasts were harvested, washed with 30 mL HS buffer, and centrifuged at 1000 g for 10 min. (brake off, 4 °C). Two methods were used to fractionate the isolated intact chloroplasts. Following the protocol proposed by Kauss et al. [37], intact chloroplasts were resuspended in 1.2 ml HS buffer and the chlorophyll concentration was adjusted to 2.0 mg Chl/ml. The appropriate amount of the chloroplast sample was centrifuged at 9300 g for 5 min., resuspended in HM buffer, kept on ice for 10 min., and then re-centrifuged at 9300 g for 5 min. The supernatant was then transferred into a new tube, and the pellet was washed 2–3 times. Following [38], the isolated intact chloroplasts were disrupted by passage through a syringe (Omnifix-F, Melsungen, Germany) with a 0.4 mm × 20 mm needle, and stroma was separated from membranes by centrifugation at 40,000 g for 10 min. Equal volumes of intact chloroplasts, membranes, and stroma were loaded onto an SDS-PAGE system, fractionated, and then analyzed after Western blotting. Primary antibodies directed against RbcL (1:10,000, Agrisera, AS03 037) and Lhcb1 (1:5,000, Agrisera, AS01 004) as markers for the different chloroplast fractions and c-Myc (1:1,000, Santa Cruz Biotechnology, sc-40) for the detection of mTERF2 were used in this study.

### 2.10. RNA sequencing (RNA-Seq) and Data Analysis

The total RNA from plants was isolated using the Direct-zol™ RNA Miniprep Plus Kit (Zymo Research, Irvine, CA, USA). RNA integrity and quality were assessed by an Agilent 2100 Bioanalyzer (Agilent, Santa Clara, CA, USA). Ribosomal RNA depletion, generation of RNA-Seq libraries, and 150-bp paired-end sequencing on an Illumina HiSeq 2500 system (Illumina, San Diego, USA) were conducted at Novogene Biotech (Beijing, China) with standard Illumina protocols. Three independent biological replicates were used per genotype. The RNA-Seq reads were analyzed on the Galaxy platform [39], as described [35]. Sequencing data have been deposited in NCBI’s Gene Expression Omnibus [40] and they are accessible through the GEO Series accession number GSE163618. 

### 2.11. GUS Staining

Histochemical staining of GUS activity was performed, as described [41]. The tissues of transgenic plants were incubated in 50 mM potassium phosphate buffer (pH 7.0) solution containing 2 mM potassium ferrocyanide (K_4_Fe(CN)_6_), 2 mM potassium ferricyanide (K_3_Fe(CN)_6_), 10 mM EDTA, 2 mM 5-bromo-4-chloro-3-indole-β-D-glucuronide, and 0.1% Triton X-100 at 37 °C overnight in the dark. The stained tissues were washed with 70% (*v*/*v*) ethanol and then with 100% (*v*/*v*) ethanol until all of the chlorophylls were removed. The GUS-stained images were observed with a light microscope (Lumar V12 microscope, Oberkochen, Germany) and then photographed with a digital camera (Canon, EOS 550D).

### 2.12. RNA Immunoprecipitation (RIP)-Seq

The sample preparation for RIP was conducted essentially as described in (Lee et al., 2017). In brief, leaves of two-week-old transgenic Arabidopsis plants expressing the mTERF2-c-myc fusion protein were vacuum-infiltrated in 1% FA (formaldehyde) for 15 min.; the fixation was stopped by additional vacuum infiltration with 125 mM glycine, and the tissue was then ground to a powder in liquid nitrogen. RNA–protein complexes were immunoprecipitated using an anti-c-myc antibody (Santa Cruz Biotechnology, sc-40), as described previously [42,43], except for slight modifications of the RIP binding buffer [20 mM HEPES (pH 7.4), 25 mM NaF, 1 mM Na_3_VO_4_, 50 mM glycerophosphate, 10 % glycerol, 500 mM NaCl, 0.5% Triton X-100, 1 mM PMSF, protease inhibitor cocktail (Roche, Germany), RNase inhibitor (Invitrogen)] and washing buffer [50 mM HEPES (pH 7.4), 500 mM NaCl, 10% glycerol, 0.05% Triton X-100]. RNA was extracted from the precipitated complexes with TRIzol reagent (Thermo Fisher Scientific) and rRNA was depleted with the RiboMinus Plant Kit for RNA-seq (Thermo Fisher Scientific) and RNA-seq libraries were generated with the NEBNext Ultra™ RNA library Prep Kit and multiplex oligos for Illumina (New England Biolabs, Ipswich, MA, USA).

### 2.13. RNA Gel-Blot Analysis

For RNA gel-blot analysis, the total RNA was extracted using Trizol (Thermo Fisher Scientific), and aliquots containing 7–10 μg of total RNA were denatured and then fractionated on a 1.2% (*w*/*v*) agarose gel and transferred to a nylon membrane (Hybond-N+; Amersham Biosciences, Freiburg, Germany). The blotted membrane was cross-linked by exposure to UV light, stained in 0.04% (*w*/*v*) methylene blue in 0.5 M sodium acetate (pH 5.2) buffer, and then used as loading control. The probes were amplified from cDNA and labeled with [α-^32^P]dCTP, probes for tRNA detection were generated by end-labeling corresponding primers with [γ-^32^P]ATP using polynucleotide T4 kinase (New England Biolabs; see Supplementary Table S1). Hybridizations were performed overnight at 65 °C (the detection of photosynthetic and *rrn* transcripts) or at 44 °C (detection of *trn*), as previously described [20]. After washing, the membranes were exposed to a phosphor imager screen, and the signals were analyzed with a phosphoimager (Typhoon; Amersham Biosciences, Freiburg, Germany) while using the program Image-Quant (GE Healthcare, Chicago, IL, USA).

### 2.14. Data Analysis

The significance of differences in chlorophyll concentration, *Fv/Fm*, mRNA expression in real-time PCR, bolting time, flowering time, leaf number, and plant height was assessed while using Student’s t-test, as described in the figure legends.

## 3. Results

### 3.1. Localisation and Tissue-Specific Expression of mTERF2

**Figure 1:**
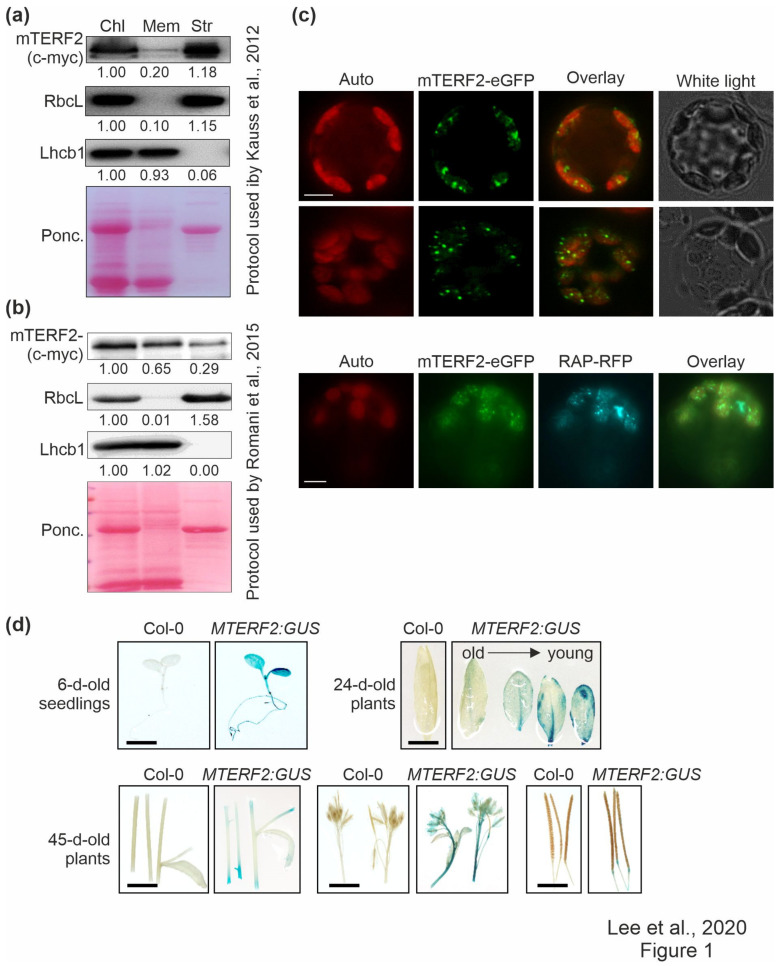
Localization and tissue-specific expression analysis of mTERF2. (**a**) and (**b**) Chloroplasts (Chl) were isolated from 3-week-old Col-0 plants overexpressing mTERF2-c-myc and fractionated into membrane (Mem) and stroma (Str) components according to (**a**) the protocol used by Kauss et al. [37], or (**b**) the protocol used by Romani et al. [20]. The fractions were subjected to SDS-PAGE, transferred to a polyvinylidene difluoride membrane (PVDF), and exposed to antibodies raised against c-myc (to detect the mTERF2-c-myc fusion protein), RbcL (as a control for the stromal fraction), or light-harvesting Chl a/b-binding protein 1 (Lhcb1; as a control for the thylakoid fraction). Quantification of signals relative to the whole chloroplast fraction (=1.00) is provided below each blotted lane. Ponc., Ponceau Red. (**c**) Transient expression of mTERF2 fused to enhanced green fluorescent protein (mTERF2–eGFP) was observed in *A. thaliana* protoplasts with fluorescence microscopy. To visualize chloroplast nucleoids, protoplasts were (co)-transformed with RAP fused to red fluorescent protein (RAP-RFP; as a marker for chloroplast nucleoids). The eGFP fluorescence is shown in green (eGFP), RFP fluorescence in cyan (RFP), autofluorescence of chloroplasts in red (Auto). Scale bars = 10 μm. (**d**) Tissue-specific expression patterns of mTERF2 were visualized with GUS activity staining in 6-day-old seedlings, leaves of 24-day-old plants, and stems, flowers, and siliques of 45-day-old plants, respectively. *MTERF2:GUS*, Col-0 plants harboring the *MTERF2* promoter fused to GUS. Bars = 1 cm.

Fluorescence microscopic analysis of guard cells in a transgenic line expressing an mTERF2–GFP fusion protein indicated that mTERF2 is targeted to chloroplasts (Babiychuk et al., 2011). However, in public databases (The Arabidopsis Information Resource, TAIR; www.arabidopsis.org), mTERF2’s localization is also annotated as mitochondrial and plasmodesmal. In order to experimentally define and investigate the intracellular localization of mTERF2 in more depth, chloroplasts from three-week-old Col-0 plants overexpressing mTERF2-c-myc were prepared and membrane- and stroma-enriched fractions were first isolated essentially according to the protocol that was used by Kauss *et al*. [37] (Figure 1a). These fractions, together with the whole chloroplast preparation, were then subjected to immunoblot analysis, and the enrichment for chloroplast sub-compartments was tested by monitoring as marker proteins stromal RbcL and thylakoid light-harvesting Chl*a*/*b*-binding protein Lhcb1. As expected, Lhcb1 was nearly exclusively detected in the membrane fraction, and RbcL mainly in the stromal fraction, a measure of the purity of the respective fractions. Using this approach, mTERF2-c-myc was mainly detected in the stromal and—to a lesser extent—in the membrane fraction (Figure 1a). Other mTERF proteins have previously been localized to nucleoids [20], a central site of PGE [44]. Nucleoid-associated proteins can be either detected mainly in the stroma [45] or associated with membranes [20], depending on the choice of the chloroplast fractionation method and the developmental status of the chloroplasts [44]. A second independent chloroplast fractionation experiment following the protocol that was used by Romani *et al*. [20] confirmed that mTERF2 is indeed associated with the membrane fraction (Figure 1b).

Therefore, in order to refine the localization of mTERF2 in the chloroplast, Col-0 protoplasts transiently overexpressing mTERF2–eGFP were examined by fluorescence microscopy, and localization to chloroplasts was confirmed (Figure 1c). Moreover, the fluorescence signal was not uniformly distributed, but appeared as small spots in the chloroplasts. Because the size and distribution of these dots were suggestive of nucleoids [46], Col-0 protoplasts were co-transformed with the RNA-binding protein RAP fused to RFP (which was previously shown to be located in nucleoids [46]), together with mTERF2–eGFP. The merging of both fluorescence signals confirmed colocalization of mTERF2 with RAP and, thus, the localization of mTERF2 to nucleoids (Figure 1c).

In order to analyze the tissue-specific expression pattern of *MTERF2*, the genomic DNA encompassing the putative *MTERF2* promoter was cloned 5’ of a GUS reporter gene, and _pro_*MTERF2*:*GUS* expression was investigated in transgenic *A. thaliana* plants. Strong GUS activity was observed in seedlings, younger leaves, and flowers, whereas weak GUS activity was observed in stems and siliques (Figure 1d).

### 3.2. Loss of mTERF2 Results in Embryo Lethality

**Figure 2:**
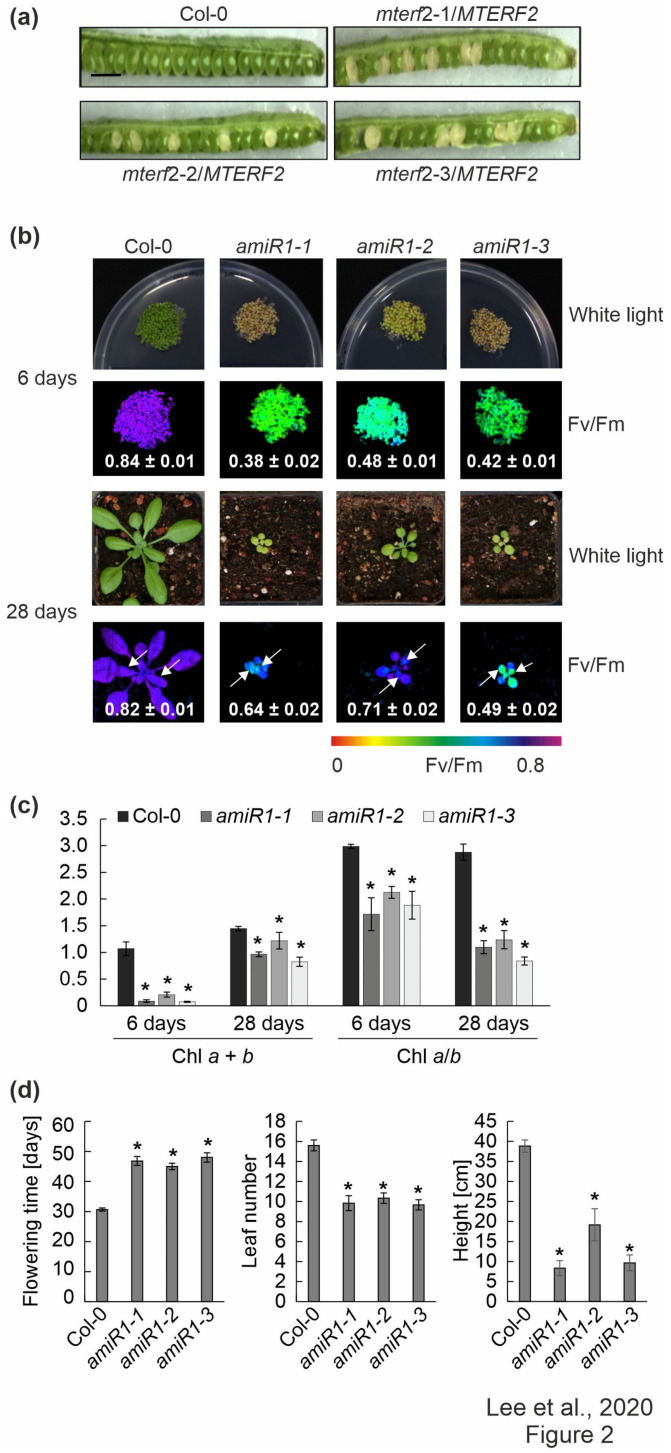
Phenotypic analysis of *mterf2* T-DNA insertion mutants and artificial microRNA (amiRNA1)-mediated *mterf2* knock-down mutants. (**a**) Seed development of *mterf2* T-DNA insertion mutants was investigated in 40-day-old wild-type (Col-0) and heterozygous mutants (*mterf2*/mTERF2). In siliques of *mterf2-1*/mTERF2, *mterf2-2*/mTERF2, and *mterf2-3*/mTERF2 plants, white ovules are found interspersed with normal green ovules. White ovules in siliques indicate aborted seeds and they account for approximately 25% of the total (44 of 143 for *mterf2-1*/*mTERF2*, 21 of 106 for *mterf2-2*/*mTERF2*, and 25 of 97 for *mterf2-3*/*mTERF2* of all the ovules analyzed). Bar = 1 cm. (**b**) Phenotypes of six-day- and 28-day-old wild-type (Col-0) and amiRNA1-generated *mterf2* mutants (*amiR1*) grown under long-day (LD) conditions (16-h light/8-h dark) on half-strength MS (+1% sucrose) media or soil, respectively. The maximum quantum yield of PSII (Fv/Fm) in the plants was measured with an Imaging PAM device. The arrows indicate the leaves for which Fv/Fm values were measured. Average values ± SD (n ≥ 6) are provided. Scale bars = 1 cm. (**c**) Acetone-extracted chlorophyll was spectrophotometrically measured and total Chl (Chl *a* + *b*) concentration and the Chl *a*/*b* ratio were calculated, as described [34]. Average values ± SD (n ≥ 4) are provided. Significant differences (*p* < 0.05) between Col-0 and mutant lines were identified while using Student’s t-test, and they are denoted by asterisks (*). (**d**) mTERF2 plays a role in plant growth and development, as evidenced by the determination of flowering time, leaf number at bolting time, and the final height of the wild-type (Col-0) and *amiR1* mutant plants. The values are represented as mean ± SD (n ≥ 6), and significant differences (*p* < 0.05) between Col-0 and mutant lines were identified by the Student’s *t*-test, and they are denoted by asterisks (*).

Three T-DNA insertion lines were identified in the SIGnAL database to obtain insight into the physiological function of mTERF2, which contains nine mTERF domains (Supplementary Figure S1a) [47]: SALK_141541 (*mterf2-1*), SALK_017427 (*mterf2-2*) and SALK_100040 (*mterf2-3*) (Supplementary Figure S1b). The PCR analysis showed that the T-DNAs are inserted in exon 1, exon 3, and intron 4 of the *AT2G21710*/*MTERF2* gene, respectively (Supplementary Figure S1b,c), but no homozygous lines could be identified. This suggested that the inactivation of the *MTERF2* gene might be lethal, which is in agreement with the notion that mTERF2 corresponds to EMBRYO DEFECTIVE 2219 [48]. The frequency of white embryos (approximately 25%) in siliques of plants heterozygous for the T-DNA insertion in *MTERF2* supports this notion (Figure 2a). More specifically, in siliques of *mterf2-1*/*MTERF2*, *mterf2-2*/*MTERF2*, and *mterf2-3*/*MTERF2* plants, we detected 44, 21, and 25 ovules that turned white among 143, 106, and 97 ovules, respectively, which is compatible with 1:3 segregation ($\chi^{2}$ = 2.54, 1.52, and 0.03, respectively), which indicated that development is disrupted in embryos that are homozygous for each of the mutant alleles. 

### 3.3. Knock-down of MTERF2 Affects Photosynthesis, Plant Growth and Development 

In order to only partially decrease *MTERF2* gene expression, independent knockdown mutants of *MTERF2* were generated with artificial microRNAs [29] targeting nucleotides 83–103 (*amiR1*, Supplementary Figure S2a) and 144–164 (*amiR2*; Supplementary Figure S3a) relative to the start codon of *MTERF2*, respectively. The *MTERF2* transcript levels were reduced to between 30% and 70% of wild-type (WT) levels, as shown by quantitative RT-PCR (Supplementary Figures S2b and S3b) and quantitative RT-PCR analysis (Supplementary Figures S2c and S3c) of six-day-old *amiR-mterf2* plants. The maximum quantum yield of PSII (F_v_/F_m_) was drastically reduced in six-day-old *amiR1-mterf2* seedlings (Figure 2b) and emerging leaves of 4-week-old soil-grown plants (Figure 2b, Supplementary Figure S3d). Moreover, when grown on soil, *amiR1-* and a*miR2-mterf2* plants displayed a reduced growth rate (Figure 2b, Supplementary Figure S3d). The pale-green coloration of cotyledons and true leaves (Figure 2b) reflected their lower overall chlorophyll (Chl) contents, and the Chl *a*/*b* ratio was significantly decreased (Figure 2c). The leaf number and height of *amiR1–mterf2* plants were reduced, and the flowering time was significantly delayed (Figure 2d).

### 3.4. Lack of mTERF2 Impairs Accumulation of Photosynthetic Proteins

**Figure 3:**
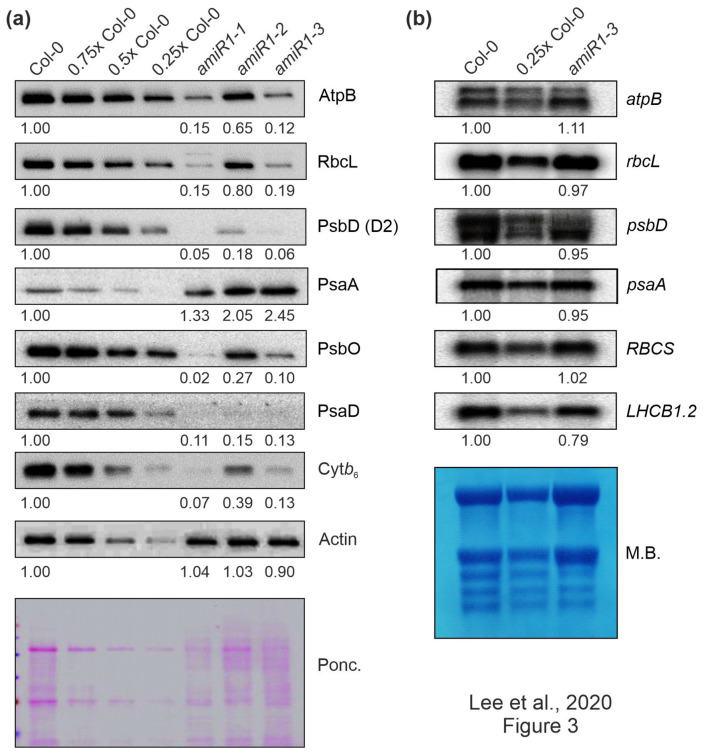
The expression of chloroplast-encoded proteins is perturbed at the posttranscriptional level in *mterf2* knock-down mutants. (**a**) Accumulation of chloroplast-encoded proteins in *mterf2* knock-down mutants. Total leaf proteins were extracted from 6-day-old wild-type (Col-0) and amiRNA1-generated *mterf2* mutants (*amiR1*), fractionated by SDS-PAGE, and blots were exposed to antibodies raised against individual photosynthetic proteins. Decreasing levels of wild-type proteins were loaded in the lanes marked Col-0, 0.75x Col-0, 0.5x Col-0, and 0.25x Col-0. Loading was adjusted to the fresh weights of leaf tissue. Actin detection and Ponceau Red (Ponc.)-staining of the blot served as loading controls. Quantification of signals relative to the wild type (= 1.00) is provided below each *mterf2* mutant lane. (**b**) Steady-state transcript levels of photosynthetic genes in wild-type (Col-0) and *amiR1–3* mutant seedlings. The total RNA was isolated from six-day-old wild-type and *amiR1–3* seedlings, and aliquots (7 μg and 3.5 μg from the wild-type; 7 μg from *amiR1–3*) were resolved on a formaldehyde-containing denaturing gel, transferred onto a nylon membrane, and then probed with [α-^32^P]dCTP-labeled complementary DNA (cDNA) fragments that are specific for transcripts encoding individual subunits of PSII (*psbD*), PSI (*psaA*), ATPase-β (*atpB*), rbcL, rbcS, and LHCII (*LHCB1.2*). Ribosomal RNAs were visualized by staining the membrane with Methylene Blue (M. B.) which served as a loading control. Quantification of signals relative to the wild type (= 1.00) is provided below each *amiR1–3* lane.

In order to determine whether the defect in photosynthetic activity in *amiR1–mterf2* plants was a consequence of the reduced accumulation of photosynthetic proteins, Western-blot analyses were performed on total protein extracts from six-day-old Col-0 and *amiR1-mterf2* seedlings. In *amiR1-mterf2* seedlings, the amounts of representative subunits of the different photosynthesis complexes were significantly reduced when compared to Col-0, except for the photosystem I (PSI) subunit PsaA (Figure 3a). Similar reductions were observed for chloroplast-encoded subunits of the ATP synthase (AtpB), PSII (PsbD), the cytochrome *b_6_ f* complex (Cyt *b_6_*), and the large subunit of RuBisCO (RbcL), as well as nucleus-encoded subunits of PSI (PsaD) and PSII (PsbO) (Figure 3a).

In contrast, Northern-blot analyses indicated that the corresponding transcripts in *amiR1–mterf2* seedlings accumulated to WT-like levels (Figure 3b). Moreover, the nuclear gene *LIGHT HARVESTING CHLOROPHYLL A/B BINDING PROTEIN1.2* (*LHCB1.2*), a marker gene for retrograde signaling [49], and *RBCS* transcripts were only slightly or not decreased at all, respectively. However, closer inspection of the Northern results indicated that amounts of chloroplast ribosomal RNAs (rRNAs) were lower in *amiR1–mterf2* seedlings, as indicated by methylene-blue staining of membranes. In the chloroplasts of land plants, the rRNAs are encoded in the *rrn* operon (Supplementary Figure S4a), which is transcribed as a single precursor molecule and processed via endonucleolytic cleavage and exonucleolytic trimming events [50]. Indeed, RNA gel-blot analyses showed that, in *amiR1–mterf2* plants, the levels of mature 16S and 23S rRNA were lower than in Col-0, while unprocessed precursors over-accumulated (Supplementary Figure S4b). Likewise, while unprocessed precursors of 4.5S rRNA also accumulated, levels of mature 4.5S and 5S rRNA were WT-like. This implies that the reduced amounts of chloroplast 16S and 23S rRNAs in *amiR1–mterf2* plants can, at least in part, account for the general reduction in chloroplast translation capacity and the delay in chloroplast development. Together, these findings suggest that the reduced accumulation of photosynthesis proteins in plants lacking mTERF2 is not caused by differential accumulation of transcripts, but implies the presence of a disturbance in another step in PGE.

### 3.5. Identification of mTERF2 Binding Sites by RIP-Seq

Altered rRNA processing can be a secondary effect, as has been observed previously for other mutants that are defective in chloroplast development [20,51,52]. In the next step of our analysis, we set out to clarify whether rRNA processing is the primary function of mTERF2. The targets of mTERF proteins could be double-stranded DNA (dsDNA) and/or RNA [10,15,16,18]. Therefore, in an attempt to identify the direct mTERF2 targets in vivo, the immunoprecipitation of nucleic acids was carried out using transgenic Col-0 plants expressing an mTERF2-c-myc fusion protein under the control of the 35S promoter (mTERF2-c-myc) and Col-0 as a control line. The expression of mTERF2-c-myc in the transgenic plants and its presence in the eluate after immunoprecipitation were confirmed by immunoblot analysis (Supplementary Figure S5a). The measurement of the amount of precipitated nucleic acids with the Qubit dsDNA and RNA high-sensitivity kits indicated that mTERF2 was likely to bind RNA (Supplementary Figure S5b). Therefore, libraries that were prepared from RNAs that co-immunoprecipitated with mTERF2-c-myc were subjected to RNA sequencing (RIP-Seq). Enrichment analysis (together with a negative control assay) identified the transfer RNAs (tRNAs) for isoleucine (*trnI.2/3*, *trnI*-GAU; 1500-fold) and alanine (*trnA.1/2*, 800-fold), and *rrn4.5S* (230-fold)—all of which are members of the *rrn* operon—as prominently enriched targets (Table 1, Supplementary Table S2). With an enrichment of 25-to 30-fold, *rrn16S* and *rrn23S* were among the weakly enriched RNAs relative to the prominently enriched targets. However, the latter result is only meaningful to a limited extent, because rRNA depletion was performed before the preparation of RNA-Seq libraries (see Materials and Methods). In addition, RNAs that correspond to 30 loci coding either for proteins that are involved in gene expression (ribosomal proteins, RNA polymerase) and photosynthesis (NADH dehydrogenase subunits A and H, PsaA and B, PsbH, AtpF and cytochrome *b_6_f* complex subunits), or associated with diverse tRNAs were moderately enriched (between 60- to 220-fold) (Table 1, Supplementary Table S2).

In *A. thaliana*, the single group I intron, eight group IIA and 12 group IIB introns are distributed between six tRNAs and 12 protein-coding genes. A closer inspection of the RNAs that were enriched by more than 60-fold in the immunoprecipitates obtained with anti-mTERF2-c-myc revealed that one, 10, and eight out of 30 targets (63%) were associated with loci whose primary transcripts contain group I, group IIA, or group IIB introns, respectively. Moreover, RNAs mapping to the downstream region of the *rps12A* exon (182-fold) and upstream region of the *rps12B* exon (223-fold) were among the most highly enriched species. These regions, intron 1A and intron 1B, represent the first and second half of the first intron in the *rps12* gene. Here, it should be pointed out that intron 1 of *rps12* (*rps12*-1) is a group IIB intron, which is transcribed from two distinct chromosomal loci, *rps12A* and *rps12B* (see Figure 6a), and then spliced together in *trans* [53].

### 3.6. RNA-Seq Supports a Role of mTERF2 in the Splicing of Group IIB Introns

**Figure 4:**
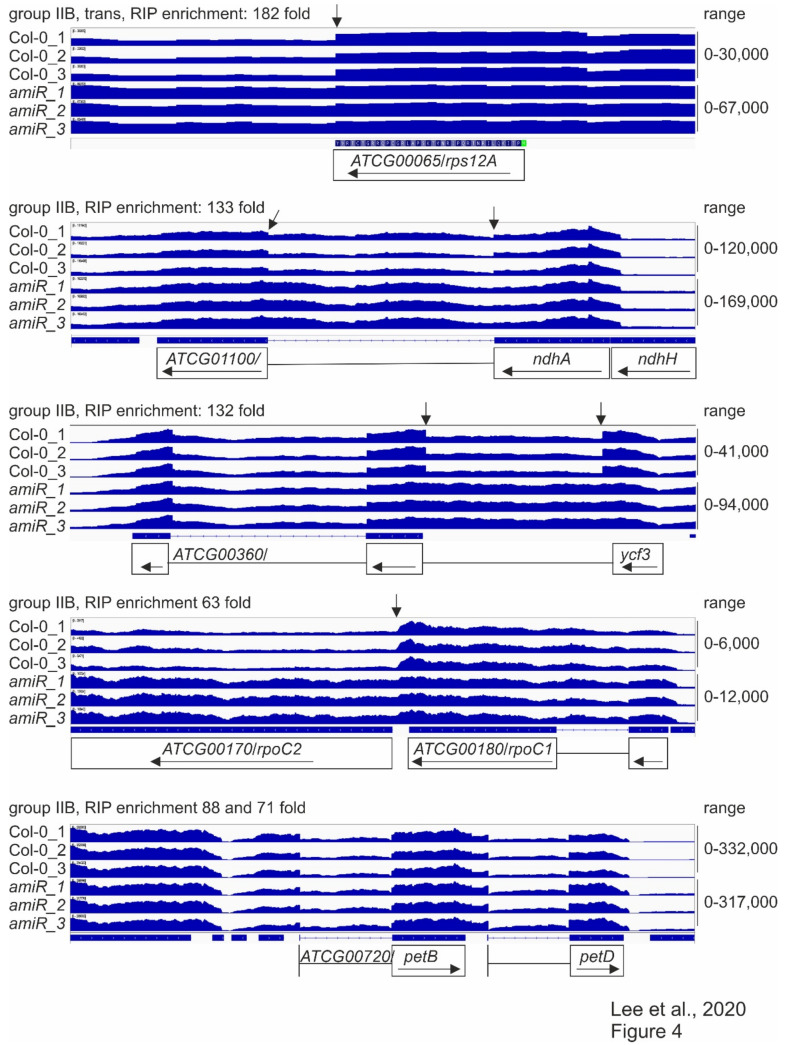
Splicing patterns of group IIB intron-containing genes.

In order to monitor the splicing status of chloroplast transcripts and obtain a general view of the RNA expression patterns when mTERF2 function is compromised, RNAs were isolated from six-day-old WT and *amiR1–mterf2* mutant seedlings grown in long-day (LD; 16 h light/8 h dark) conditions, and then subjected to long non-coding (lnc)RNA sequencing. With this method, the levels of *atpB* (1.60-fold)*, rbcL* (0.99-fold)*, psbD* (0.85-fold)*, psaA* (1.88-fold) mRNAs—which had already been investigated by RNA gel-blot analysis (see Figure 3)—were only slightly altered and, notably, not down-regulated (Supplementary Table S3). In fact, among the plastid-encoded transcripts, only *YCF6* levels were reduced by more than two-fold, while the expression levels of 59 plastid genes rose more than two-fold. Moreover, the presence of reduced levels of *MTERF2* mRNA in the *amiR1–mterf2* mutant were verified (Supplementary Figure S6) and confirmed the fidelity of the RNA-Seq data. Therefore, coverage files of the sequencing data were used to determine the status of putative mTERF2 target loci that were identified by RIP-Seq analysis with the help of the Integrative Genomics Viewer (IGV; [54]) (for a summary, see Table 1, Supplementary Table S2). Note that this method does not provide any information on the status of the three most highly enriched targets (*trnI.3*, *trnA.1*, *rrn4.5S*): Because of the many modifications of tRNAs, their structure, and their small size, our RNA library preparation method for genome-wide RNA-Seq analysis does not allow for them to be mapped in a representative manner. Moreover, before library preparation for whole-genome RNA-Seq, RNAs were depleted of rRNA. However, RNA gel-blot analyses showed that, although there was no difference in the levels of mature 4.5S rRNA levels, an unprocessed precursor over-accumulated in *amiR1–mterf2* plants (see Supplementary Figure S4). With regard to protein-encoding loci, we found that, in particular, the splicing of group IIB introns in *ycf3*-1, *ndhA*, *rpoC1*, and *rps12*-1 was affected (Figure 4, column “IGV” in Table 1).

RNA-Seq analysis was conducted, as described in the Materials and Methods. The read depths of *rps12A*, *ndhA*, *ycf3*, *rpoC1,* and *petB* transcripts detected in wild-type (Col-0) and *amiR1–3*-*mterf2* mutant (*amiR*) seedlings were visualized with the Integrative Genomics Viewer (IGV). While differences in splicing pattern or read-through (indicated by vertical arrows) can be seen for *rps12A*, *ndhA*, *ycf3* and *rpoC1*, the splicing of *petB* is similar in Col-0 and *amiR1–3*-*mterf2* mutant seedlings.

Taken together, these RNA-Seq data, together with identified binding sites by RIP-Seq, point to a role for mTERF2 in the splicing of group IIB introns.

### 3.7. mTERF2 Promotes ycf3-1 and rps12-1 Splicing

**Figure 5:**
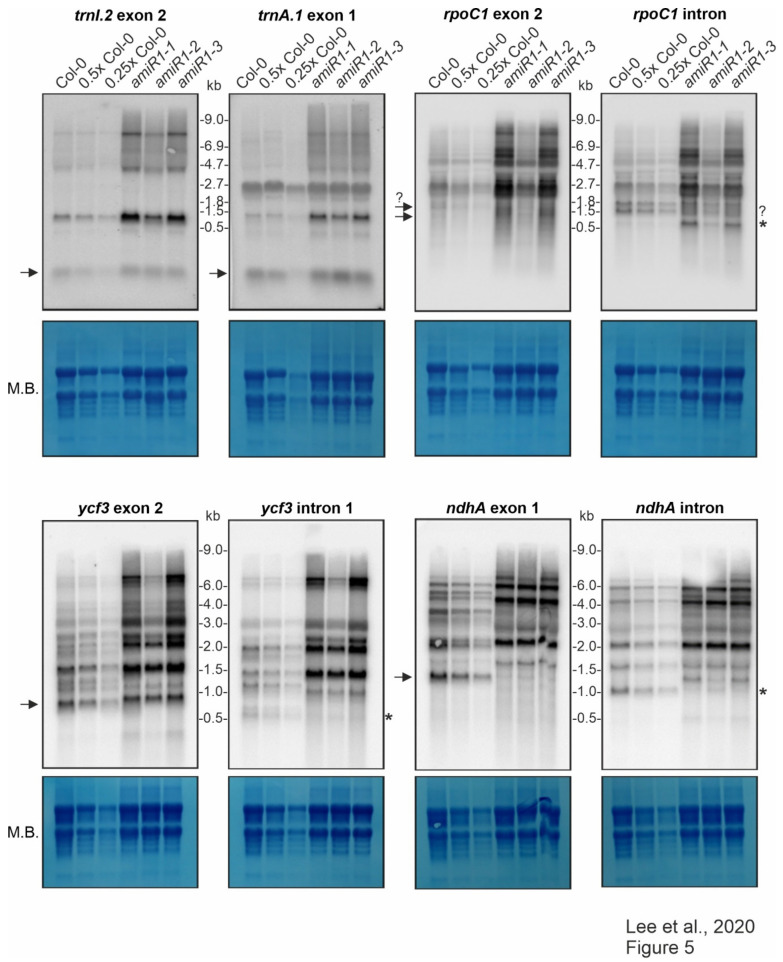
Expression and processing of chloroplast *trnI.2*, *trnA.1*, *rpoC1*, *ycf3*, and *ndhA* in wild-type (Col-0) and *mterf2* knock-down mutant plants.

RNA gel-blot analyses were first carried out with probes targeting the exons of *trnI.2/3*, *trnA.1/2* in order to investigate in greater depth whether mTERF2 affects the splicing of putative targets. The *amiR1–mterf2* mutant seedlings indeed accumulated more of the unprocessed precursors than did the WT; nevertheless, the mature forms of these tRNAs were present in essentially WT levels (Figure 5). This indicates that lack of mTERF2 does not prohibit the splicing of *trnI.2/3* and *trnA.1/2*.

The splicing of the group IIB intron-containing transcripts *ycf3*, *ndhA*, *rpoC1*, and *rps12* was assayed with exon- and intron-specific probes. For *ycf3*-1 and *ndhA*, the results showed a large increase in the abundance of unspliced precursors and the total absence of the corresponding mature forms in *amiR1–mterf2* mutant lines (Figure 5).

The levels and splicing patterns of *trnI.2, trnA.1, rpoC1, ycf3* and *ndhA* were analyzed in six-day-old wild-type (Col-0) and *mterf2* mutant plants *(amiR1–1, amiR1–2, amiR1–3)*. The total RNA was isolated from six-day-old seedlings, and aliquots [10 μg (Col-0), 5 μg (0.5x Col-0), and 2.5 μg (0.25x Col-0)) from the wild-type; 10 μg from *mterf2* mutants)] were resolved on a formaldehyde-containing denaturing gel, transferred onto a nylon membrane, and then probed with [γ-^32^P]ATP end-labeled oligonucleotide probes specific for the second exon of *trnI.2* and first exon of *trnA.1*, and [α-^32^P]dCTP-labeled cDNA fragments that are specific for *rpoC1*, *ycf3*, and *nhdA* exons and introns, as indicated. Ribosomal RNA was visualized by staining the membrane with Methylene Blue (M. B.) as a loading control. The arrows or the asterisks (*) in the blots indicate mature transcripts or the excised intron, respectively, and the question-mark indicates uncertainty regarding the size of the mature transcript or intron, respectively.

**Figure 6:**
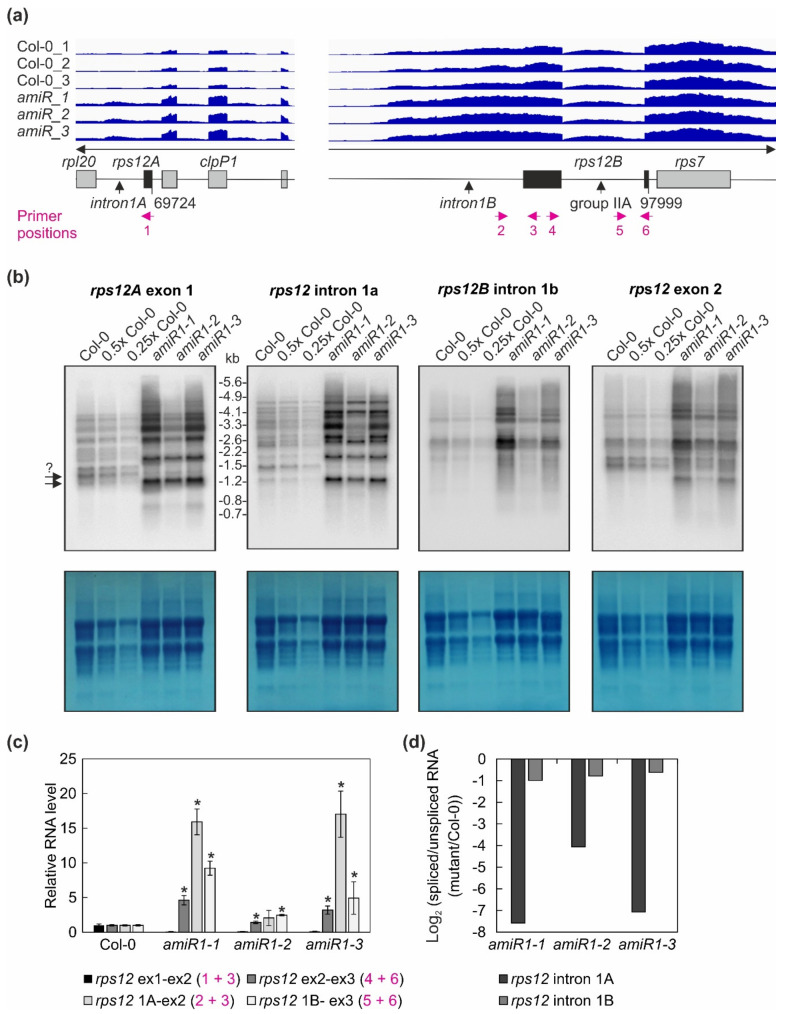
The expression and processing of chloroplast *rps12* transcripts in wild-type (Col-0) and *mterf2* mutant plants. (**a**) RNA-Seq analysis was performed with RNA extracted from wild-type (Col-0) and *amiR1–3*-*mterf2* mutant (*amiR*) seedlings. The read depths of *rps12A* and *rps12B* transcripts detected in Col-0 and *amiR* seedlings were visualized with the Integrative Genomics Viewer (IGV). The schematic representations of the chloroplast *rps12* genes below the IGV snapshots depict the *rps12A* and *rps12B* genes, which are approximately 28 kb apart in the chloroplast genome, and have to be trans-spliced. The *rps12A* gene containing exon 1 and intron 1A is cotranscribed with *rpl20* and *clpP1* transcripts and the *rps12B* gene containing intron 1B, exon 2, intron 2l and exon 3 is cotranscribed with *ndhB* and *rps7*. The magenta arrows indicate the primer positions used in panel (**c**). (**b**) The levels and splicing patterns of *rps12* transcripts were analyzed in six-day-old wild-type (Col-0) and *mterf2* mutant plants *(amiR1-1, amiR1-2, amiR1-3)*. Total RNA was isolated, and aliquots resolved on a formaldehyde-containing denaturing gel were transferred onto a nylon membrane and probed with [α-^32^P]dCTP-labeled cDNA fragments specific for *rps12* exon 1, intron 1A, intron 1B, and exon 2. rRNA was visualized by staining the membrane with Methylene Blue (M. B.) as a loading control. The arrows with the question mark indicate uncertainty about the size of the mature transcript. (**c**) Quantitative RT-PCR analysis of 6-day-old wild-type (Col-0), *amiR1-1, amiR1–2* and *amiR1-3* mutant seedlings. PCR was conducted with primer pairs marked in panel (**a**) and *AT4G36800*, encoding an RUB1–conjugating enzyme (RCE1) as a control. The RNA expression levels are reported relative to that in the Col-0, which was set to 1. Bars indicate standard deviation (SD). Significant differences between mutants and Col-0 were evaluated with Student’s *t*-test (*p* < 0.05) and are denoted by the asterisks (*). (**d**) The ratios of spliced to unspliced *rps12* transcripts in *amiR1–1, amiR1-2*, *amiR1-3,* and Col-0 were calculated based on the result shown in panel (**c**), and the data are displayed as log_2_ ratio of spliced to unspliced *rps12* transcripts in the mutants compared to the wild-type (Col-0).

Small transcripts that were recognized by the intron-specific probe in the WT (indicated by asterisks in Figure 5) may represent excised introns; a small portion of the *ndhA* intron is detected in the weaker *amiR1–2-mterf2* mutant. However, defective *ndhA* splicing has also been detected in *A. thaliana* and rice mutants lacking PPR4, a protein that is specifically required for *rps12*-1 splicing, and this effect has been discussed as a possible secondary consequence of reduced translation [55]. In seedlings that were grown on Norflurazon, an inhibitor of phytoene desaturase, rRNAs are depleted (see Figure 3 in [56]) Mapping of published RNA-Seq data derived from Col-0 seedlings grown on control medium and on medium supplemented with this inhibitor [57] also showed defective *ndhA* splicing (Supplementary Figure S7). The role of mTERF2 in the splicing of the *rpoC* and *rps12* introns was also not completely clear from our RNA gel-blot data. Although, in both cases, unspliced precursors significantly accumulated when mTERF2 was lacking (Figure 5 and Figure 6b), it was difficult to judge whether the spliced form was indeed missing. This is because a transcript of similar (but not exactly the same) size as the mature form was also detected in *amiR1–2-mterf2* mutant seedlings (Figure 5 and Figure 6b).

**Figure 7:**
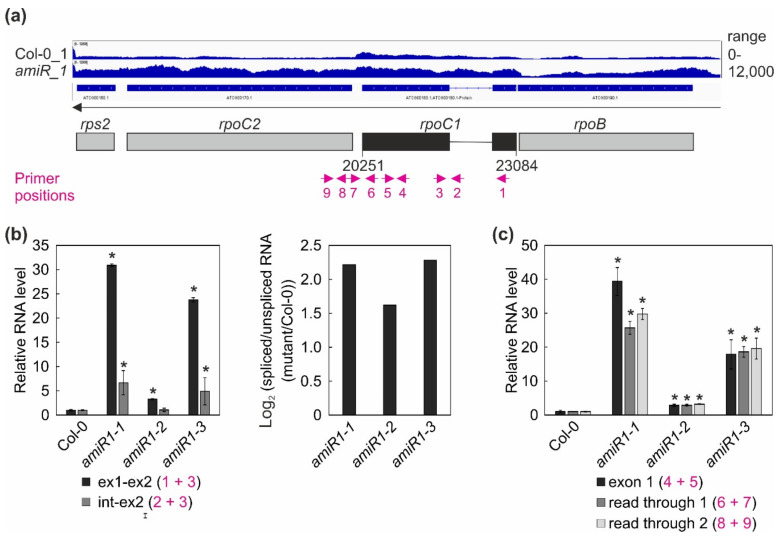
Expression and processing of chloroplast *rpoC1* transcripts in wild-type (Col-0) and *mterf2* mutant plants. (**a**) RNA-Seq analysis was performed with RNA extracted from wild-type (Col-0) and *amiR1-3*-*mterf2* mutant (*amiR*) seedlings. The read depths of *rps2*, *rpoC2*, *rpoC1*, and *rpoB* transcripts detected in Col-0 and *amiR* seedlings were visualized with the Integrative Genomics Viewer (IGV). Below the IGV data, a schematic representation of the chloroplast *rpoC1* gene is shown and the magenta arrows indicate the primer positions used in Figure (**b**). (**b**,**c**) Quantitative RT-PCR analysis of RNAs isolated from 6-day-old Col-0 and *amiR1–mterf2* mutant plants (*amiR1-1, amiR1-2,* and *amiR1-3*). PCR was conducted with the primer pairs marked in panel (**a**) and *AT4G36800*, encoding an RUB1–conjugating enzyme (RCE1) as a control. The RNA expression levels are reported relative to that in the Col-0, which was set to 1. The ratios of spliced to unspliced *rpoC1* transcript between *amiR1–mterf2* mutant seedlings (*amiR1-1, amiR1-2,* and *amiR1-3)* and wild-type (Col-0) were calculated based on the relative levels of *rpoC1* exon 1, exon 2, and the intervening intron, and the data are depicted as the log_2_ ratio of spliced to unspliced *rpoC1* transcript in the *amiR1–mterf2* mutant plants compared to Col-0 in (**b**). Relative RNA levels of exon 1, read through 1 and -2 in Col-0 and *amiR1–mterf2* mutant plants are shown in (**c**). Bars indicate standard deviations (SD). Significant differences between mutants and Col-0 were evaluated with Student’s *t*-test (P < 0.05) and they are denoted by the asterisks (*).

Moreover, IGV data (i) did not provide a very clear indication of the status of *rpoC1* splicing either in WT or in *amiR1–mterf2* mutant lines; indeed, the splicing of this transcript appeared to be enhanced in *amiR1–mterf2* mutant seedlings, and (ii) these data suggested read through of *rpoC1* in *amiR1-mterf2* mutant seedlings. In an attempt to unequivocally monitor the splicing status of *rps12* and *rpoC1*, and/or a potential read through of *rpoC1*, quantitative RT-PCR analyses was performed with primers indicated in Figure 6a and Figure 7a. 

Overall, this analysis revealed a clear decrease in the ratio of spliced to unspliced RNAs for the trans-spliced group IIB intron 1 of *rps12*, while the splicing of *rps12* intron 2 (a group IIA intron) was only marginally affected (Figure 6c,d). This reduction in splicing efficiency was not due to the perturbed accumulation of the *rps12* pre-mRNAs, since they actually accumulated to greater than WT levels (Figure 4, Figure 6). For *rpoC1*, quantitative RT-PCR analysis confirmed the enhanced splicing of *rpoC1* when mTERF2 is lacking (Figure 7b). Moreover, different primer combinations used to detect read-through events showed 20- to 40-fold higher RNA levels 3′ of *rpoC1* in the stronger *amiR1-1*- and *amiR1-3-mterf2* alleles, while they were also significantly, but only three-fold, higher in the weaker *amiR1-2-mterf2* mutant (Figure 7c). The amounts of read-through transcripts were comparable to those that were detected for the first exon of *rpoC1*.

To summarize, a direct function in *ndhA* splicing cannot be unequivocally assigned to mTERF2, as defects in the splicing of this transcript are also observed as a secondary effect in mutants with ribosomal or developmental defects. However, the fact that group IIB intron splicing of *ycf3*-1 and *rps12-1* was impaired in *amiR1–mterf2* mutant plants, together with the RIP-Seq data, strongly suggests that mTERF2 directly promotes the splicing of at least these introns. 

## 4. Discussion

Plants code for approximately 30 mTERF family members [12]. Although the molecular function of only five of them had been characterized so far [10,14,15,16], it has become clear that the mTERF proteins play a broad role in organellar gene expression. In this study, we have assigned both a function and RNA substrates to mTERF2. We have shown that mTERF2 is an essential protein that is localized to chloroplast nucleoids, where it associates with *ycf3* and *rps12* RNAs to mediate group IIB intron splicing.

Introns are classified into two main families, group I and group II, depending on their conserved primary and secondary structures, as well as differences in their splicing mechanisms [24]. Ancient, retromobile group II introns consist of an RNA and a protein-encoding component. The RNA is a ribozyme (catalytic RNA) that is capable of self-splicing in vitro, while the protein component contains endonuclease, reverse transcriptase (RT), and maturase domains [58]. Group II introns have attracted much attention due to their putative relationship with eukaryotic nuclear pre-mRNA introns and the spliceosome [6,25,27]. Thus, the central domain of Prp8, which is the most highly conserved protein in the spliceosome, is related to the catalytic domain of the RT [59], and a small nuclear RNA (snRNA) in the spliceosome adopts a group II intron-like tertiary conformation to catalyze splicing [60]. Group II introns could have been incorporated into eukaryotic genomes either during the same endosymbiotic event that gave rise to mitochondria and chloroplasts [25,27] or by subsequent migrations [25]. The approximately 20 group II introns each found in the chloroplast and mitochondrial genomes of angiosperms have become immobile, their RNA structures have degenerated, and all except two have completely lost the intron-encoded protein domain. Therefore, in the context of nuclear-organellar coevolution, the development of efficient splicing in organelles was accompanied by the emergence of nucleus-encoded RNA-binding proteins [58,61,62]. The first such proteins affected a subset of chloroplast splicing events and, in maize, the discovery of CHLOROPLAST RNA SPLICING1 (CRS1) and CRS2 opened up a new research area—the elucidation of the mechanisms that regulate organellar RNA splicing [63]. Gradually, it became apparent that splicing in chloroplasts involves many more proteins that belong to various families—including maturases [6], and members of RNA splicing and ribosome maturation (CRM), pentatricopeptide repeat (PPR), DEAD-box RNA helicase (DBRH), Plant Organelle RNA Recognition (PORR), and ACCUMULATION OF PHOTOSYSTEM ONE (APO) families. In 2004, the mTERF family was added to this list by the discovery that maize mTERF4 promotes splicing of at least *trnI*-GAU, *trnA*-UGC, and *rpl2* [22]. Some splicing factors, especially those from the PPR family, are specific for a single chloroplast intron, whereas others aid in splicing of multiple introns, which are usually structurally related [25,58,64]. Based on RNA sequence structure and mechanism [27], group II introns are divided into three major groups. Group IIC introns have only been discovered in eubacteria, while groups IIA and IIB are found in flowering plants [25,27]. Each of the introns in angiosperm chloroplasts is known to require at least one nucleus-encoded protein for its splicing [58]. Here, we have shown that mTERF2 mediates at least the splicing of *ycf3* intron 1 (*ycf3*-1) and *rps12* intron 1 (*rps12*-1), both of which belong to group IIB.

### 4.1. Splicing of ycf3

The *ycf3* gene harbors two introns, *ycf3*-1 and *ycf3*-2. APO1 [65] and the PPR proteins OTP51 [66] and THA8 [67] are required for the splicing of *ycf3*-2. Five proteins have been shown to participate in the splicing of *ycf3*-1. CRS2, a protein that is related to a peptidyl-tRNA hydrolase enzyme, facilitates the splicing of nine group II introns, including *ycf3*-1 [68]. The two CRM family members CAF1 and CAF2 bind to CRS2 to form the CRS2–CAF1 and CRS2–CAF2 complexes. These two complexes are involved in the splicing of a set of group II introns, but they have two substrates in common—the *ycf3*-1 and *ndhA* introns [69]. CFM2 promotes the splicing of the *ycf3*-1, *trnL*, *ndhA*, and *clpP*-2 introns [70]. In contrast to the aforementioned splicing factors, the PPR protein PHOTOSYSTEM I BIOGENESIS FACTOR2 (PBF2) is specifically required for *ycf3*-1 intron splicing, but it also cooperates with CAF1 and CAF2 [71]. With mTERF2, we have now identified a sixth factor that is involved in *ycf3*-1 splicing.

The *ycf3* gene codes for a member of the tetratricopeptide repeat (TPR)-like superfamily that is required for PSI assembly and stability. Thus, Ycf3 cooperates with the nucleus-encoded thylakoid protein Y3IP1 in the assembly of PSI in tobacco and Arabidopsis [72]. A lack of OTP51, APO1, or PBF2 consequently results in a failure to accumulate the PSI complex [65,66,71]. Moreover, *otp51* and *pbf2* mutant plants can only grow heterotrophically and in dim light [66,71]. Interestingly, in *amiR1–mterf2* seedlings, amounts of the plastid-encoded PSI subunit PsaA actually increased (see Figure 3), while the PsaA protein is undetectable in both *pbf2* [71] and *otp51* [66] mutant seedlings. However, the PsaD protein is virtually undetectable in *amiR1–mterf2* seedlings (see Figure 3). Importantly, PsaA and PsaB assemble at an early stage of PSI biogenesis to form a pre-complex, including Ycf3, which is required for the subsequent integration of PsaC-J [73]. In a double mutant for the two nuclear genes encoding PsaD, none of the investigated subunits of PSI were detected, which demonstrated that PsaD is essential for the stability of the complex [74]. Therefore, the fact that PsaA accumulates in *amiR1–mter2* seedlings is surprising, although it should be noted that the levels of PsaA present in PsaD knock-out plants were not investigated [74].

### 4.2. Splicing of rps12-1 

Some group II intron sequences in plant mitochondria and chloroplasts have been split through into two or more segments that are encoded in distant parts of the respective genomes, owing to genomic rearrangements [25]. The "discontinuous group II intron" form of trans-splicing, which is found in plant and algal chloroplasts and plant mitochondria, involves the joining of spatially separated, transcribed coding sequences, presumably through interactions between intronic RNA segments [75], which then physically associate to form a tertiary structure that resembles a typical group II intron [25]. In plant chloroplasts, only *rps12*-1, which is a group IIB intron, is trans-spliced [75] (see Figure 6); the second intron in the *rps12* pre-mRNA is a group IIA intron. In *amiR*-*mterf2* plants, the splicing of the *rps12*-1 intron is affected, while the splicing of *rps12*-2 is essentially normal (see Figure 6). Factors that are known to be required for *rps12*-1 splicing are the CRS2–CAF2 complex, which is required for the splicing of the five group IIB introns of *ndhB*, *petB*, *ndhA*, *ycf3*-1, and *rps12*-1 [58], and the DEAD-box RNA helicase RH3, which promotes the splicing of *trnI*, *trnA*, *rpl2*, and both *rps12* introns [76]. Apart from these three splicing factors, each of which acts on several substrates, the PPR proteins EMB2394 and PPR4, and the ribosomal protein uL18–L8, are thought to be specifically required for *rps12*-1 trans-splicing in *A. thaliana* [55,77,78] and, in the case of PPR4, also in maize [79] and rice [55]. It has been proposed that the binding of EMB2654 and PPR4 to *rps12* intron 1a and intron 1b, respectively, is needed to fold the RNA into the structure that is necessary for the formation of the splicing complex. Moreover, this likely involves the RNA chaperone activity of PPR4 [55]. The uL18-L8 protein is a member of the uL18 ribosomal protein family originally dedicated to the binding of 5S rRNAs to incorporate them into ribosomes. Recently, it was discovered that two of the eight members of this family have repurposed their RNA-binding capacity to associate with the introns whose removal they promote [78].

Maize and rice *ppr4* mutants are seedling-lethal [55,79], and *A thaliana mterf2* (see Figure 2), *ppr4* [55], and *emb2394* [77] mutants are embryo-lethal, highlighting the indispensability of Rps12 and chloroplast translation for the survival of both dicot and monocot plants.

### 4.3. Splicing of ndhA

Splicing of the *ndhA* transcript requires the action of the CRS2–CAF1 and CRS2–CAF2 complexes [69] and CFM2 [70], as in the case of the *ycf3*-1 intron. The homologous PPR proteins PpPPR_66 and PPR66L are also involved in *ndhA* splicing in *Physcomitrella patens* and *A. thaliana*, respectively [80]. In rice, the *white stripe leaf4* (*wsl4*) mutant, which is defective in a PPR protein, disrupts the splicing of *ndhA, atpF, rpl2*, and *rps12*-1 during early leaf development [81]. However, further studies are needed in order to clarify whether splicing is indeed the primary function of WSL4 and, moreover, the functions of the *A. thaliana* and maize WSL homologs are unknown. In addition, a mutation in the Arabidopsis PPR gene *PIGMENT-DEFICIENT MUTANT 1* (*PDM1*), which is also known as *SEEDLING LETHAL 1* (*SEL1*) [82], resulted in the defective splicing of *ndhA*, *trnK*, and *rps12*-2 introns [83]. However, the PDM1/SEL1 protein has previously been shown to be an RNA editing factor for *accD* sites [82], and the binding of this protein to the respective introns has yet to be been shown. Therefore, *ndhA* splicing is probably especially prone to directly or indirectly triggered translational imbalances, such as those seen in *pdm1*/*sel1* mutants [82,83] and in *ppr4* mutants, in which *ndhA* splicing is also secondarily affected [55]. This theory is supported by the effect of norflurazon on *ndhA* splicing. The growth of seedlings on norflurazon, which is a carotenoid biosynthesis inhibitor and is not implicated in splicing, causes *ndhA* splicing deficiencies that are similar to those seen in the absence of mTERF2 (see Supplementary Figure S7). Therefore, defective *ndhA* splicing in *amiR-mterf2* seedlings, although it is very clear and pronounced (see Figure 5), must nevertheless be evaluated with caution, and does not unambiguously prove that mTERF2 is an *ndhA* splicing factor in spite of the fact that RNAs matching *ndhA* were co-immunoprecipitated with mTERF2 in our RIP-Seq assay (see Table 1).

### 4.4. Molecular Functions of mTERFs

It is now clear that mTERFs evolved to bind DNA as well as RNA. mTERF5 [17,18] and mTERF8 [19] are examples of mTERFs binding to DNA, which bind different dsDNA sequences that are associated with the *psbEFLJ* polycistron. The levels of plastid-derived transcripts are not substantially altered in an *mterf8* mutant, except for the *psbJ* transcript, which is specifically increased [19]. Indeed, the loss of mTERF8 leads to a dramatic 30- to 50-fold rise in transcription 3′ of the *psbJ* gene, which indicates transcriptional read-through. The binding of mTERF8 to the anticipated 3′ termination region of *pbsJ* has been confirmed by electrophoresis mobility shift assay (EMSA) and chromatin immunoprecipitation (ChIP) analyses, and the transcription-termination activity of mTERF8 was confirmed in an in-vitro system [19]. The mTERF5 protein, on the other hand, binds to the +30 to +51 region of the *psbEFLJ* polycistron [17,18]. mTERF5 acts as a transcriptional pausing factor by binding to this region and inducing pausing of the plastid-encoded RNA polymerase (PEP) complex, according to Ding et al. [17]. Subsequently, additional PLASTID TRANSCRIPTIONALLY ACTIVE 6 (pTAC6) is recruited by mTERF5, which enhances PEP activity, and boosts *psbEFLJ* transcription. The binding of mTERF5 to single-stranded DNA and RNA at this site has been experimentally excluded [17]. Métegnier et al. [18] identified the 5’ region of the *ndhA* gene as a second binding site for mTERF5. Furthermore, they detected a strong reduction in the levels of small RNAs derived from the 5′ terminus of the *psbE* and *ndhA* genes in the *mterf5* mutant. These small RNAs might represent footprints that are protected against exonuclease action by binding to PPRs [84]. Therefore, the authors concluded that mTERF5 binds to these regions in order to stimulate transcription as well as promote the stabilization of the 5′-ends of processed *psbE* and *ndhA* transcripts.

The mTERF6 protein binds in vivo to plastid isoleucine transfer RNA (*trnI.2*), the gene for which is located in the ribosomal RNA (rRNA) operon, and it promotes the maturation, but not splicing, of this tRNA [20]. *In vitro*, recombinant mTERF6 can bind to both RNA and dsDNA, and it terminates transcription at a site that is located in the intron of *trnI.2*. However, the termination function of this site could not be substantiated in vivo, and, more recently, Zhang et al. [21] reported a transcription termination function of mTERF6 downstream of the plastid *rpoA* polycistron. We detected 20- to 40-fold higher RNA levels 3′ of *rpoC1* in the stronger *amiR1–1*- and *amiR1–3-mterf2* alleles (see Figure 7), which suggests a termination function of mTERF2 at this site, but this remains to be confirmed.

With mTERF2 (this work), mTERF4 [13,22], and mTERF15 [23], three mTERF members have now been placed firmly in a splicing context. In maize, mTERF4 co-immunoprecipitates with introns and known splicing factors to promote at least the splicing of the group IIA introns of *trnI.2*, *trnA*, *rpl2*, and *atpF*, as well as *ycf3*-2, a group IIB intron. Additionally, in the *A. thaliana mterf4* mutant, the introns in *atpF* and *rpl2*, and the group IIa introns of *rps12*-2 and *clpP*-2, are not spliced out [13]. However, failure to remove the first three of these introns is also observed in wild-type plants grown on spectinomycin, an inhibitor of organelle translation, while the second intron of *clpP* is correctly spliced under these conditions. Furthermore, the splicing of this last intron is thought to occur independently of MatK, which otherwise acts as a trans-acting splicing factor for group IIA introns [85]. This suggests a direct role for BSM in splicing out the second *clpP* intron, although binding to this region has not yet been shown. The second intron of *clpP* is not found in maize, but the intron splicing function of mTERF4 is conserved in the *A. thaliana* and maize. The introns that are spliced by mTERF2 are conserved in maize, and it remains to be seen whether maize mTERF2 is also a splicing factor. In *mterf15*, the splicing of *nad2* intron 3 is significantly reduced, a defect that is fully rescued in the complemented plants, which suggests that mTERF15 might participate in splicing of the mitochondrial *nad2* transcript [23]. However, the investigation of splicing in *mterf15* mutants was primarily motivated by the role of mTERF4 in chloroplast intron splicing, and the splicing defect in *mterf15* could also be secondarily caused, because the binding of mTERF15 to the target remains to be shown. 

The functions that are summarized above indicate that some mTERFs might be specific for a single target (*A. thaliana* mTERF8), while others can bind to several targets [maize mTERF4, *A. thaliana* mTERF2, mTERF5, and mTERF6]. Moreover, one mTERF can serve different functions (mTERF5, mTERF6). In this context, the question arises as to whether mammalian mTERFs are also involved in splicing. However, until recently it was thought that, in contrast to the plant mitochondrial and plastid genomes, the mammalian mitochondrial genome does not contain any introns. This view was challenged when introns were detected in mammalian mitochondrial transcripts by next-generation sequencing technology [86]. Although the authors postulate that those introns are spliced by the nuclear spliceosome machinery, which is imported into the mitochondria, a splicing function of mammalian mTERFs cannot be excluded.

## 5. Conclusions

With one exception, all of the proteins that are required for organellar gene expression (OGE) in land plants are encoded in the nucleus. Progress in uncovering the functions of these proteins provides insights into the molecular innovations that emerged during the coevolution of nuclear and organellar genomes. Thus, studies have revealed that gene expression in plant organelles is even more complicated than first expected, and it involves a still growing number of nucleus-encoded proteins from diverse families. The mTERF proteins that have been characterized so far also participate either in aspects of OGE that are not typical of the nuclear/cytosolic compartment (e.g., group II intron splicing), or in processes characteristic for bacteria that have not previously been shown to take place in plant organelles (e.g., transcriptional pausing). It has become evident that some mTERFs are essential (mTERF1, mTERF2, mTERF6), the lack of others (mTERF5, mTERF8, mTERF9, mTERF15, mTERF18, mTERF22) provokes milder phenotypes, some of which (mTERF10, mTERF11) only become pronounced under adverse environmental conditions [15,33]. Changes in OGE occur in response to a changing environment, and perturbations of OGE homeostasis regularly result in the activation of acclimation and tolerance responses [87]. Thus, the investigation of the molecular functions of mTERFs will provide deeper insights into the complex OGE machineries, and it will also add to our understanding of how changes in OGE enable plants to acclimate.

## Figures and Tables

**Table 1:** Top-ranking loci (> 90-fold enrichment) that were identified in mTERF2 RIP-Seq experiments.

Gene ID	Fold Enrichment	Gene model Description	Gene Symbol	Intron Classification	IGV
ATCG01200	1487	tRNA-Ile	*trnI.3*	group IIA	Maybe not informative ^1^, therefore Northern performed
ATCG00940	826	tRNA-Ala	*trnA.1*	group IIA	Maybe not informative ^1^, therefore Northern performed
ATCG00960	233	4.5S ribosomal RNA	*rrn4.5S.1*	/	Maybe not informative ^2^, therefore Northern performed
ATCG01230	223	30S RIBOSOMAL PROTEIN S12B.	*rps12B*	group IIA/group IIB	Higher accumulation, especially first intron and upstream (intron 1b), splicing of groupII A intron OK
ATCG01310	194	50S RIBOSOMAL PROTEIN L2	*rpl2.2*	group IIA	Higher accumulation, splicing OK
ATCG00065	182	30S RIBOSOMAL PROTEIN S12A.	*rps12A*	group IIB	Higher accumulation downstream (intron 1a), trans-splicing defect?
ATCG00400	159	tRNA-Leu	*trnL.1*	group I	Higher accumulation
ATCG01190	154	tRNA-Ala	*trnA.2*	group IIA	^1^
ATCG01150	142	tRNA-Arg	*trnR.3*	/	^/^
ATCG01100	133	NADH dehydrogenase ND1	*ndhA*	group IIB	Splicing defect
ATCG00360	132	Encodes a protein required for PSI assembly and stability	*ycf3*	group IIB	First intron splicing defect
ATCG00750	131	30S RIBOSOMAL PROTEIN S11	*rps11*	/	/
ATCG00830	106	50S RIBOSOMAL PROTEIN L2	*rpl2.1*	group IIA	Higher accumulation, splicing seems to be OK
ATCG00670	91	PLASTID-ENCODED CLP P.	*pclpP*	group IIA	Splicing OK, but carefully look downstream (ATCG0065, RPS12A)

Highlighted in bold are those loci which show a splicing defect in RNA-Seq data (see Figure 4). ^1^ Because of the large number of modifications found in tRNAs, their structure, and their small size, the RNA library preparation method used for genome-wide RNA-Seq analysis does not allow them to be mapped in a representative – and informative – manner. ^2^Of limited relevance, since rRNA depletion was performed prior to library preparation of immunoprecipitated RNAs. IGV, Integrated Genomics Viewer (http://software.broadinstitute.org/software/igv/).

## Supplementary Materials

The following are available online at https://www.mdpi.com/2073-4409/10/2/315/s1, Supplementary Figure S1: Identification of *mterf2* T-DNA insertion mutants, Supplementary Figure S2: Generation of artificial microRNA (amiRNA)-mediated *mterf2* knock-down mutants (*amiR1*), Supplementary Figure S3: Generation and phenotypic analysis of a second set of artificial microRNA (amiRNA)-mediated *mterf2* knock-down mutants (*amiR2*), Supplementary Figure S4: Expression and processing of chloroplast rRNAs in wild-type (Col-0) and *mterf2* knock-down seedlings, Supplementary Figure S5: Confirmation of the presence of mTERF2-c-myc and detection of nucleic acids in the eluate after coimmunoprecipitation, Supplementary Figure S6: Confirmation of RNA-Seq data, Supplementary Figure S7: The splicing of *ndhA* is also disturbed under norflurazon conditions, Supplementary Table S1: Primers used in this study, Supplementary Table S2: Enrichment values of the RIP (RNA-immunoprecipitation)-Seq experiment, Supplementary Table S3: Transcript level changes of plastid-encoded genes in 6-day-old LD-grown *amiR1–3-mterf2* seedlings compared to Col-0.
