# Supplementary material for: Arabidopsis Mitochondrial Transcription Termination Factor mTERF2 Promotes Splicing of Group IIB Introns
Source: Cells. 2021 Feb 3;10(2):315. doi: 10.3390/cells10020315 (PMC7913559; doi:10.3390/cells10020315)
Supplement: Supplementary file 1 [file cells-10-00315-s001.zip › Supplementary Figures.pdf]

cells MDPI

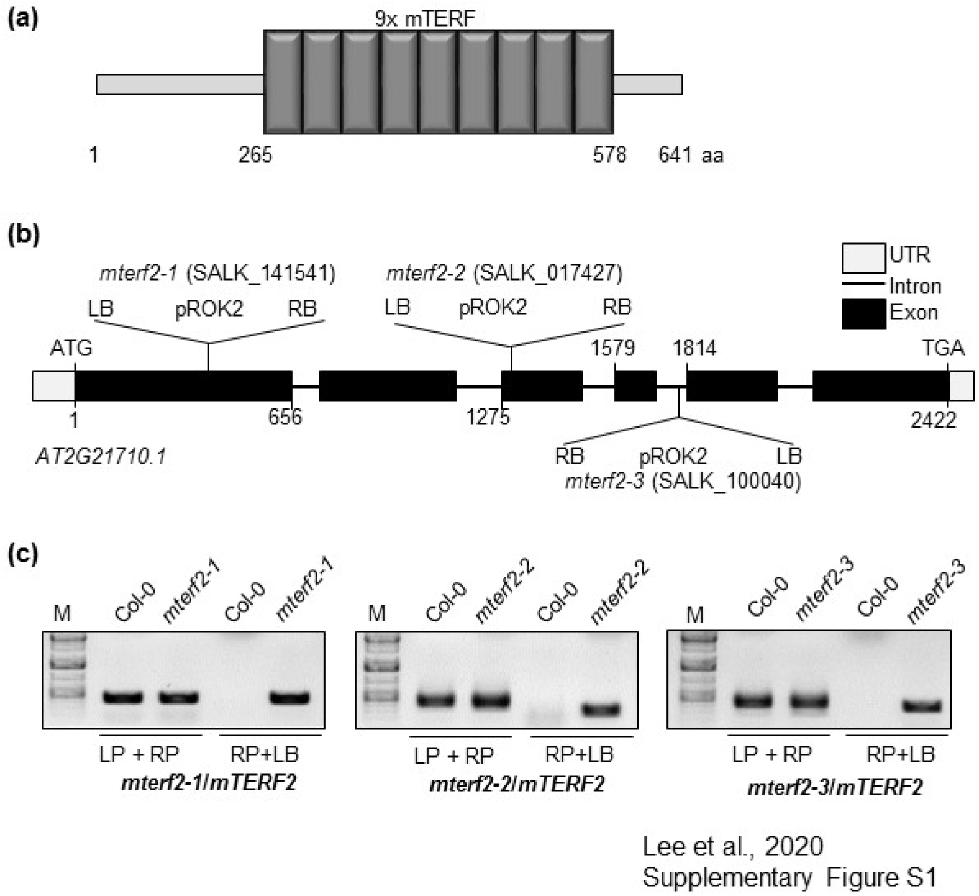

Supplementary Figure S1. Identification of *mterf2* T-DNA insertion mutants.

(a) Schematic representation of the mTERF2 domain structure. The number of mTERF motifs in mTERF2 is indicated by rectangles and their locations are indicated by amino acid (aa) positions in the mTERF2 protein.

(b) Schematic representation and T-DNA tagging of the *MTERF2* (*AT2G21710*) locus. Exons (black boxes), introns (black lines) and the 5′ and 3′ UTRs (grey boxes) are shown. Numbers are given relative to the start codon ATG. Locations and orientation of T-DNA insertions are indicated. Note that the insertions are not drawn to scale.

(c) Confirmation and identification of homozygous T-DNA insertions in the different *mterf2* mutant lines. The gene-specific left and right primers (LP and RP) were used for amplification of sequences around the T-DNA insertion, and RP was used together with the T-DNA left-border primer (LB) for verification of the T-DNA insertion.

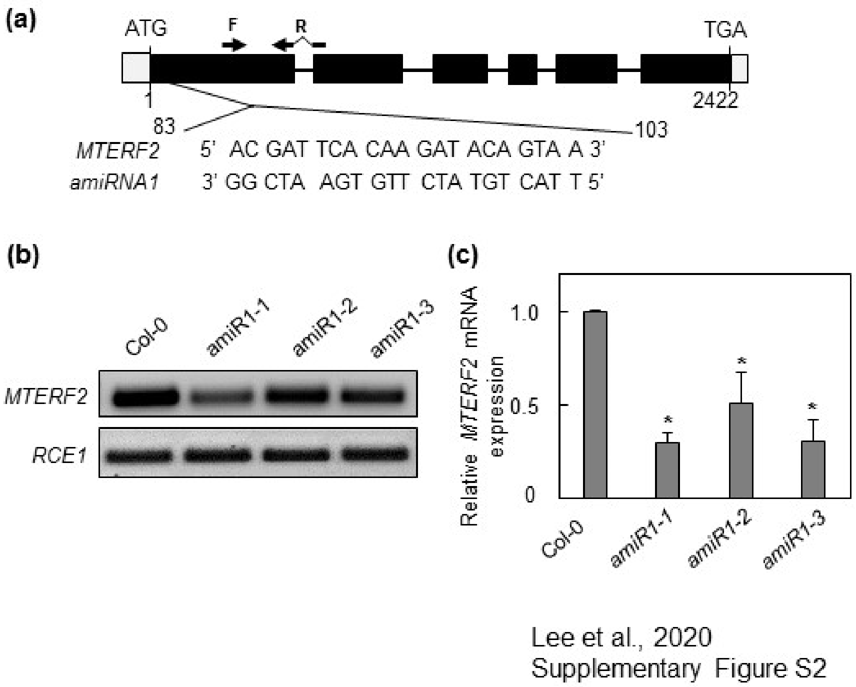

Supplementary Figure S2. Generation of artificial microRNA (amiRNA)-mediated *mterf2* knock-down mutants (*amiR1*).

(a) Schematic representation of the artificial microRNA (amiRNA) and its target sequence. Nucleotide sequences of *amiRNA1* and its wild-type *MTERF2* target are shown.

(b) RT-PCR analysis of 6-day-old wild-type (Col-0) and three selected *amiR1 mTERF2* knock-down mutant plants. RT-PCR was performed with primers specific for the *AT2G21710* splice form 1 (*MTERF2.1*) and *AT4G36800*, encoding a RUB1-conjugating enzyme (RCE1), served as a control.

(c) Quantitative RT-PCR analysis of *MTERF2.1* mRNA expression in 6-day-old amiR1-mediated *mterf2* knock-down mutant plants. *MTERF2.1* mRNA levels are expressed relative to those in the Col-0 control, which was set to 1. The results were normalized to the expression level of *AT4G36800* (*RCE1*). Bars indicate standard deviations.

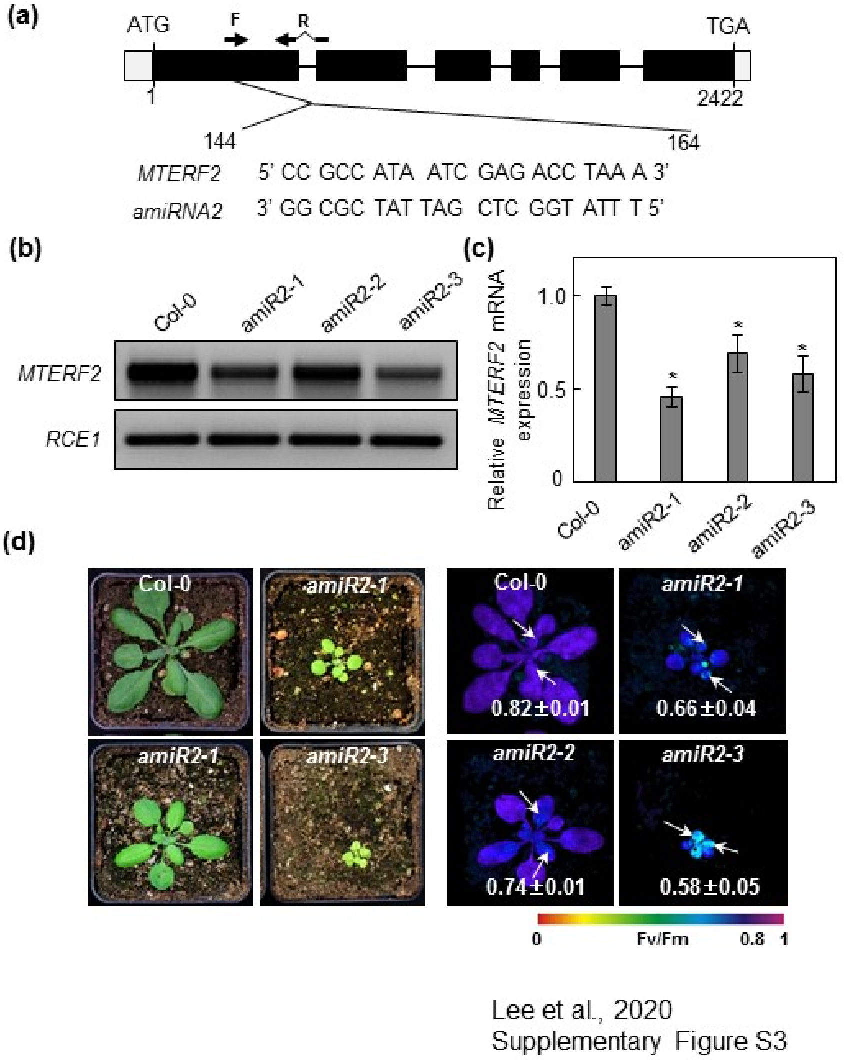

Supplementary Figure S3. Generation and phenotypic analysis of a second set of artificial microRNA (amiRNA)-mediated *mterf2* knock-down mutants (*amiR2*).

(a) Schematic representation of the artificial microRNA (amiRNA) and its target sequence. Nucleotide sequences of *amiRNA2* and its wild-type *MTERF2* target are shown.

(b) RT-PCR analysis of 6-day-old wild-type (Col-0) and three selected *amiR2 mTERF2* knock-down mutant plants. RT-PCR was performed with primers specific for the *AT2G21710* splice form 1 (*MTERF2.1*) and *AT4G36800*, encoding a RUB1-conjugating enzyme (RCE1) as a control.

(c) Quantitative RT-PCR analysis of *MTERF2.1* mRNA expression in 6-day-old amiR1-mediated *mterf2* knock-down mutant plants. *MTERF2.1* mRNA levels are expressed relative to those in the Col-0 control which was set to 1. The results were normalized to the expression level of *AT4G36800* (*RCE1*). Bars indicate standard deviations (SDs).

(d) Phenotypes of 28-day-old wild-type (Col-0) and *amiR2* mutants grown on soil under LD conditions (16-h light/8-h dark). The maximum quantum yield of PSII (Fv/Fm) was measured via Imaging PAM. The arrows indicate the leaves of which Fv/Fm values were measured. Average values ± SD (n ≥ 6) are provided. Scale bars = 1 cm.

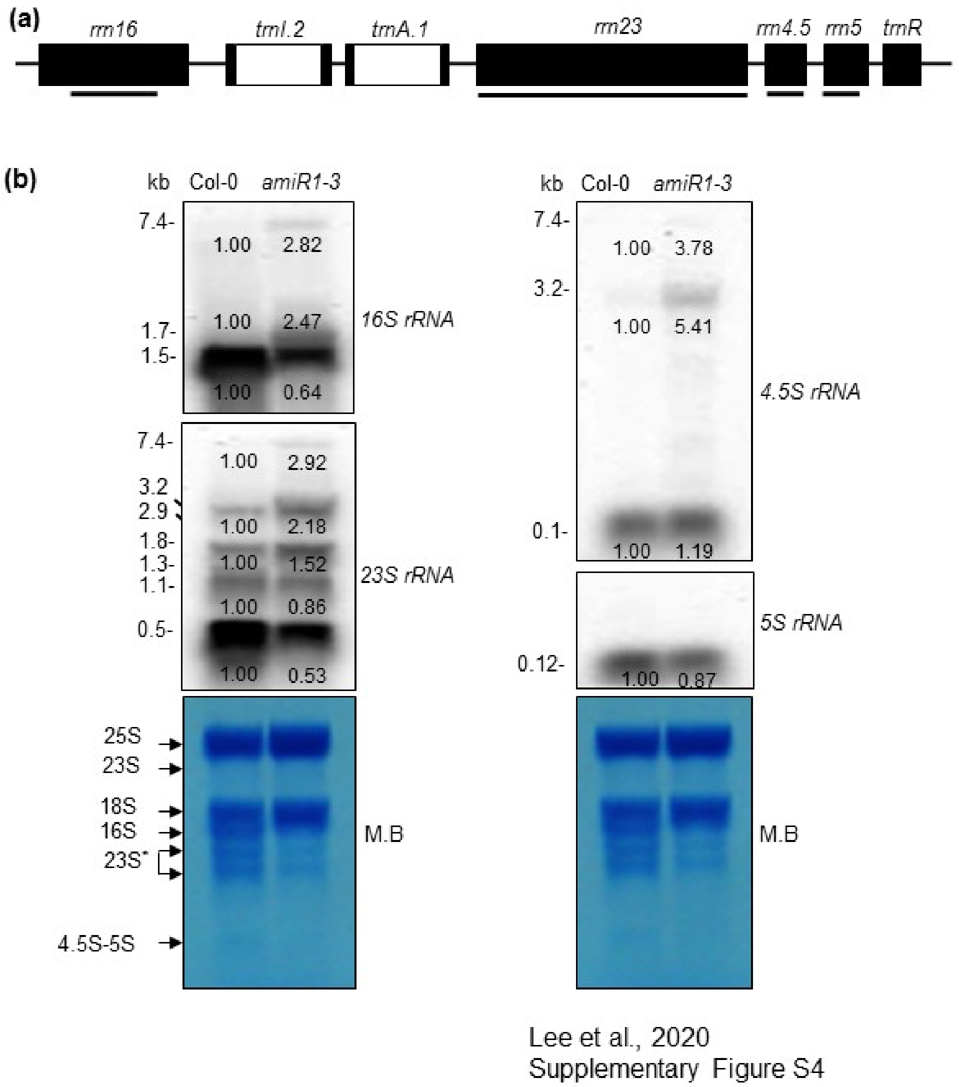

Supplementary Figure S4. Expression and processing of chloroplast rRNAs in wild-type (Col-0) and *mterf2* knock-down seedlings.

(a) Schematic representation of the chloroplast rRNA operon. Black rectangles represent exons genes encoding rRNAs and tRNAs, and white rectangles represent introns, respectively. The positions of the probes used for Northern blot analyses are indicated by black solid lines below the operon structure.

(b) Analysis of transcript levels and splice forms of *rrn* genes. Total RNA was isolated from 6-day-old wild-type (Col-0) and *amiR1-3* mutant seedlings, resolved on a formaldehyde-containing denaturing gel, transferred onto a nylon membrane, and probed with [α-^{32}P]dCTP-labeled cDNA fragments specific for *rrn16* and *rrn23*, and with [γ-^{32}P]ATP end-labeled oligonucleotide probe specific for *rrn4.5* and *rrn5*. rRNA was visualized by staining the membrane with Methylene Blue (M. B.) as a loading control.

(a)

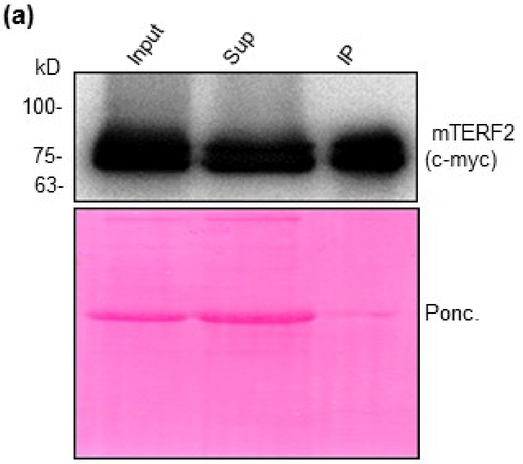

(b)

	dsDNA high sensitivity	RNA high sensitivity
Sample	ng/uL	ng/uL
WT	0.0373	Too low
IP mTERF2	0.0788	1.04

Lee et al., 2020
Supplementary Figure S5

Supplementary Figure S5. Confirmation of the presence of mTERF2-c-myc and detection of nucleic acids in the eluate after coimmunoprecipitation.

(a) Col-0 plants expressing an mTERF2-c-myc fusion protein under the control of the 35S promoter (mTERF2-c-myc) were subjected to an RIP-Seq experiment as described in Materials and Methods. Proteins extracted from the input, the supernatant (Sup) and the eluate (IP) were fractionated by SDS-PAGE, and blots were exposed to antibodies raised against c-myc. Ponceau Red (Ponc.)-staining of the blot served as a loading control.

(b) The amounts of nucleic acids present in the eluate were determined with the Qubit dsDNA and RNA high sensitivity kits, respectively.

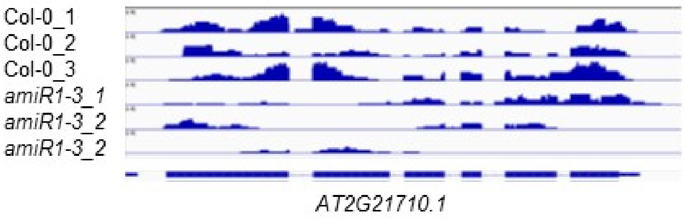

Lee et al., 2020
Supplementary Figure S6

Supplementary Figure S6. Confirmation of RNA-Seq data.

The RNA-Seq experiment was performed like described in Materials and Methods. The read depths of *AT2G21710* (*mTERF2*) transcripts detected in wild-type (Col-0) and *amiR1-3-mterf2* mutant (*amiR1-3*) seedlings were visualized with the Integrative Genomics Viewer (IGV).

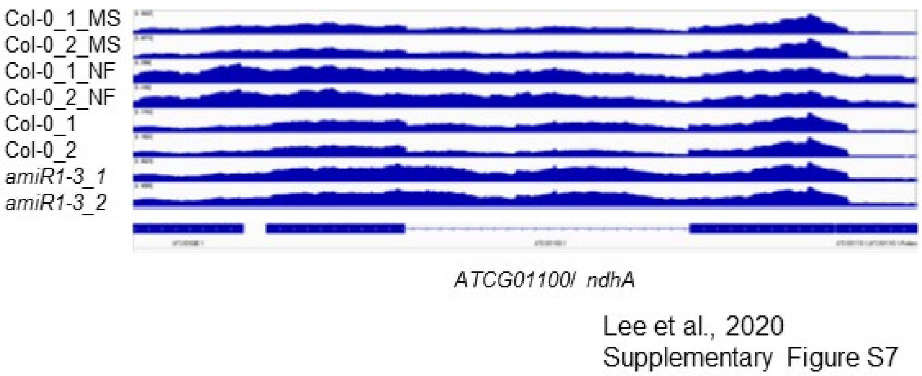

Supplementary Figure S7. The splicing of *ndhA* is also disturbed under norflurazon conditions. The reads from an RNA-Seq experiment performed in were mapped to the TAIR10 genome. The depths of reads mapping to *ndhA* in untreated Col-0 seedlings (Col-0_MS), norflurazon-treated (Col-0_NF), and of our Col-0 and *amiR1-3-mterf2* mutant (*amiR1-3*) seedlings were visualized with the Integrative Genomics Viewer (IGV).
