# Supplementary material for: Arabidopsis Mitochondrial Transcription Termination Factor mTERF2 Promotes Splicing of Group IIB Introns
Source: Cells. 2021 Feb 3;10(2):315. doi: 10.3390/cells10020315 (PMC7913559; doi:10.3390/cells10020315)
Supplement: Supplementary file 1 [file cells-10-00315-s001.zip › Supplementary Table S1.pdf]

Supplementary Table S1. Primers used in this study.

Atg number	Description	Primer sequence (from 5′ to 3′)
**Genotyping**		
AT2G21710	*mterf2-1* RP1	GCGCTTTAACTATCATCTCCCTC
	mterf2-1 LP1	GCTACTACACCTCAACGTTTTCC
	mterf2-2 RP2	TGAGTATCTAGAGAGCAATGGTGTG
	mterf2-2 LP2	TGACTAGAGGCTTCCATCTTTCT
	mterf2-3 RP3	GGAAATCAGCAATCATTTCGCCT
	mterf2-3 LP3	TTGCAGGAGATGGGTATCCCTAA
**Fluorescence localization**		
AT2G21710	*mTERF2.1* cDNA amplified with attB1 and attB2 overhangs	GGGGACAAGTTTGTACAAAAAAGCAGGCTATGCTTCTCCACTGCAACGT<br>GGGGACCACTTTGTACAAGAAAGCTGGGTGTTCTGTCAAATCCTCTTCT
**GUS promoter assay**		
AT2G21710	*mTERF2.1* promoter region amplified with attB1 and attB2 overhangs	GGGGACAAGTTTGTACAAAAAAGCAGGCTAAGTTATTTCTGTCTGCTTTTA<br>GGGGACCACTTTGTACAAGAAAGCTGGGTGTTTAGAGCAACGTTGTCGGAGAGAA
**Artificial microRNA (amiRNA) construction**		
mterf2-amiR1	I:	gaTTACTGTATCTTGTGAATCGGtctctcttttgtattcc
	II:	gaCCGATTCACAAGATACAGTAAtcaaagagaatcaatga
	III:	gaCCAATTCACAAGAAACAGTATtcacaggtcgtgatatg
	IV:	gaATACTGTTTCTTGTGAATTGGtctacatatatattcct
mterf2-amiR2	I:	gaTTTAGGTCTCGATTATCGCGGtctctcttttgtattcc
	II:	gaCCGCGATAATCGAGACCTAAAtcaaagagaatcaatga
	III:	gaCCACGATAATCGACACCTAATtcacaggtcgtgatatg
	IV:	gaATTAGGTGTCGATTATCGTGGtctacatatatattcct
pDONR207-amiR	A:	GGGGACAAGTTTGTACAAAAAAGCAGGCTCAAACACACGCTCGGACG
	B:	GGGGACCACTTTGTACAAGAAAGCTGGGTCATGGCGATGCCTTAAATA
mTERF2 RT-PCR and real-time		TGGTTAGGAAAGGTAGATTTGGGA<br>TGCTTCAACCACCTTACAAGA
**Real-time PCR**		
AT4G36800	"Housekeeping" gene RCE1	CTGTTCACGGAACCCAATTC<br>GGAAAAAGGTCTGACCGACA

AT3G45140	*LOX2*	ATTCAAAGGCAAGCTCCAAT<br>AACACCCATTCCGGTAACAC
AT1G72520	*LOX4*	CCGTCATCACCACCATCATCTG<br>CGTAGCCACCGTAAGGATATTGT
AT3G49620	*DIN11*	TGGATCACCTTATGAGTTTGAG<br>CGTCAACAAGCCATAGTCAG
AT3G27690	*BBX29*	CTGTGAGTCTTGTTTAGCTCGT<br>GTTCTCCTCATCGGAGAGGT
ATCG00065	*rps12* exon1	ATCCGAAACGTCACGAAATC
ATCG00905/ ATCG01230	*rps12* intron1b	TCTCACACCGGGTAAATCCT
ATCG00905/ ATCG01230	*rps12* exon2	GGAGCCGTATGAGGTGAAAA
ATCG00905/ ATCG01230	*rps12* exon2	CGTAAAGTTGCCAGAGTACGA
ATCG00905/ ATCG01230	*rps12* intron 2	TTTGGCTTTTTGACCCCATA
ATCG00905/ ATCG01230	*rps12* exon 3	TGTGGAAAGCCGTATTCGAT
ATCG00180	*rpoC1* exon 1	ATGGTATTTGAAACGTCTTCCTAG
ATCG00180	*rpoC1* exon 2	ATTCAAATGAACCTCGTAATCGTAA
ATCG00180	*rpoC1* intron 1	AACTACTTGAGCCGGATGAGA
ATCG00180	read-thr_*rpoC1*-1_F	GTGGAAGGACGCACTATTT
ATCG00180	read-thr_*rpoC1*-1_R	TCTCCAATAFCTGGAGATAAG
ATCG00180	read-thr_*rpoC1*-2_F	GAACCACAGTTGGTCATATTTC
ATCG00180	read-thr_*rpoC1*-2_R	GGGTGTCCTAGAATTCCTTT
ATCG00180	read-thr_*rpoC1*-3_F	CTCAAACTCATTGTCGAATCC
ATCG00180	read-thr_*rpoC1*-3_R	CCATCCCGAAGTGATCTATT
**RNA gel blot analysis**		
ATCG00270	*psbD*	ATGACTATAGCCCTTGGTAA<br>AACCAGATTGACCTAGTGGA
ATCG00350	*psaA*	TGGGCTAAACCCGGTCATTT<br>GGAACCAACCAGCAAAAAGC
ATCG00480	*ATPβ*	TTAGGTCCTGTCGATACTCG<br>ACCCAATAAGGCGGATACCT
ATCG00490	*rbcL*	CGTTGGAGAGACCGTTTCTT<br>CAAAGCCCAAAGTTGACTCC
AT5G38410	*rbcS*	ATGGCTTCCTCTATGCTCTCTC<br>AATGAAGGCGCCCGGGTACTCC
AT1G29910	*LHCB1.2*	ATGGCCGCCTCCACAATGGCT<br>CCTCGGCCTCTCCCAACGGCC
ATCG00920	*rrn16S*	TTTAAGTCCGCCGTCAAATC<br>TCAACGGTGGCAACTAAACA
ATCG00950	*rrn23S*	TAATACGACTCACTATAGGGAGATTCA<br>AACGAGGAAAGGCTTACG<br>AGGAGAGCACTCATCTTGG
ATCG00960	*rrn4.5S*	GATGCCTCAGCTGCATACATCACTGCAC

		TTCCACTTGACACCTATCGTAATGATAA
		ACGGCTCGTCTCGCCGTGACCTTC
ATCG00970	*rrn5S*	CCTGGCGTCGAGCTATTTTTCCGCAGGA
		CCTCCCCTACAGTATCGTCACCGCAGTA
		GAGTTTAACCACCAAGTTCGGGAT
ATCG00930	*trnI.2/3,* second exon	ATTTGAACCAGAGACCTCGC
ATCG00930	*trnI.2/3,* intron	CCCAGGCACAACGACGCAA
ATCG00940	*trnA.1*	GAGCGGAGCTCTACCAACTG
ATCG00065	*rps12* exon1	ATGCCAACCATTAAACAACT
		ATACACCCGAGTACATGTTC
ATCG00065	*rps12* intron1a	GTGCGACTCGTTTAGATCATAG
		TCGTGTAAGCCGTGAATGAG
ATCG00905/	*rps12* exon2	ACTATCACCCCCAAAAAACC
ATCG01230		AACGCCCTTGTTGACGATCC
ATCG00905/	*rps12* intron1b	ATAACCGCATGGATAAGCTC
ATCG01230		AGATAAGTCACCCTTACTGC
ATCG00360	*ycf3* exon2	CGGATGTCGGCTCAATCTGAAGG
		AGGGGTTTCGTTCTAATGCCCGA
ATCG00360	*ycf3* intron1	AGGGGAAGAAGCACTACGCCG
		ACGGCTCCTCCTTTAGGTGCAT
ATCG01100	*ndhA* exon1	ATTGGACCTGAATACGCCGG
		AGGAGCAATACTTGAGATGGCA
ATCG01100	*ndhA* intron	CGATTCAGAGTATGCTCCTATCCACCGA
		C
		CAGAACCGTACATGAGGTCTTGGC
ATCG00180	*rpoC1* exon2	TTTTCTTTTGCTAGGCCCATAACT
		TTAGGTATCATATGAACAGG
ATCG00180	*rpoC1* intron	TGTGATTTGATCGAAAT
		ATTGGGATAGGGTCCACT
